# Ji-Ni-De-Xie ameliorates type 2 diabetes mellitus by modulating the bile acids metabolism and FXR/FGF15 signaling pathway

**DOI:** 10.3389/fphar.2024.1383896

**Published:** 2024-05-21

**Authors:** Yiwen Tao, Fang Peng, Lijie Wang, Jiayi Sun, Yin Ding, Shuangfeng Xiong, Ugen Tenzin, MiMa, Tsedien Nhamdriel, Gang Fan

**Affiliations:** ^1^ State Key Laboratory of Southwestern Chinese Medicine Resources, School of Ethnic Medicine and School of Pharmacy, Chengdu University of Traditional Chinese Medicine, Chengdu, China; ^2^ Innovative Institute of Chinese Medicine and Pharmacy, Chengdu University of Traditional Chinese Medicine, Chengdu, China; ^3^ Dege County Tibetan Hospital (Institute of Tibetan Medicine), Dege, China; ^4^ Department of Tibetan Medicine, University of Tibetan Medicine, Lhasa, China; ^5^ Meishan Hospital of Chengdu University of Traditional Chinese Medicine, Meishan, China

**Keywords:** Tibetan medicine, Ji-Ni-De-Xie, bile acid metabolism, FXR/FGF15 signaling, type 2 diabetes mellitus

## Abstract

**Introduction:** Ji-Ni-De-Xie (JNDX) is a traditional herbal preparation in China. It is widely used to treat type 2 diabetes mellitus (T2DM) in traditional Tibetan medicine system. However, its antidiabetic mechanisms have not been elucidated. The aim of this study is to elucidate the underlying mechanism of JNDX on bile acids (BAs) metabolism and FXR/FGF15 signaling pathway in T2DM rats.

**Methods:** High-performance liquid chromatography-triple quadrupole mass spectrometry (HPLC-QQQ-MS) and UPLC-Q-Exactive Orbitrap MS technology were used to identify the constituents in JNDX. High-fat diet (HFD) combined with streptozotocin (45 mg∙kg^−1^) (STZ) was used to establish a T2DM rat model, and the levels of fasting blood-glucose (FBG), glycosylated serum protein (GSP), homeostasis model assessment of insulin resistance (HOMA-IR), LPS, TNF-α, IL-1β, IL-6, TG, TC, LDL-C, HDL-C, and insulin sensitivity index (ISI) were measured to evaluate the anti-diabetic activity of JNDX. In addition, metagenomic analysis was performed to detect changes in gut microbiota. The metabolic profile of BAs was analyzed by HPLC-QQQ-MS. Moreover, the protein and mRNA expressions of FXR and FGF15 in the colon and the protein expressions of FGF15 and CYP7A1 in the liver of T2DM rats were measured by western blot and RT-qPCR.

**Results:** A total of 12 constituents were identified by HPLC-QQQ-MS in JNDX. Furthermore, 45 chemical components in serum were identified from JNDX via UPLC-Q-Exactive Orbitrap MS technology, including 22 prototype components and 23 metabolites. Using a T2DM rat model, we found that JNDX (0.083, 0.165 and 0.33 g/kg) reduced the levels of FBG, GSP, HOMA-IR, LPS, TNF-α, IL-1β, IL-6, TG, TC, and LDL-C, and increased ISI and HDL-C levels in T2DM rats. Metagenomic results demonstrated that JNDX treatment effectively improved gut microbiota dysbiosis, including altering some bacteria (e.g., *Streptococcus* and *Bacteroides*) associated with BAs metabolism. Additionally, JNDX improved BAs disorder in T2DM rats, especially significantly increasing cholic acid (CA) levels and decreasing ursodeoxycholic acid (UDCA) levels. Moreover, the protein and mRNA expressions of FXR and FGF15 of T2DM rats were significantly increased, while the expression of CYP7A1 protein in the liver was markedly inhibited by JNDX.

**Discussion:** JNDX can effectively improve insulin resistance, hyperglycemia, hyperlipidemia, and inflammation in T2DM rats. The mechanism is related to its regulation of BAs metabolism and activation of FXR/FGF15 signaling pathway.

## Graphical Abstract

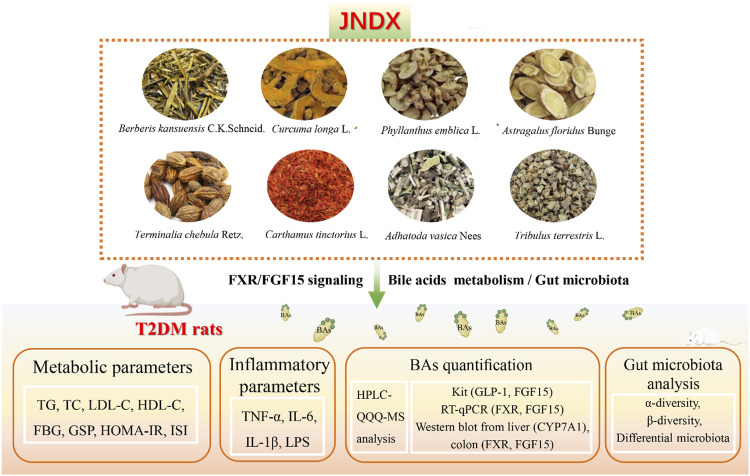

## 1 Introduction

Diabetes mellitus is a chronic metabolic disease characterized by hyperglycemia primarily caused by deficiency of insulin secretion and utilization (Wu et al., 2022). Currently, diabetes mellitus has a high incidence and serious complications, which seriously affects the quality of life of patients. Type 2 diabetes mellitus (T2DM) accounts for 90% of diabetes cases. The major characteristics of T2DM are insulin resistance and hyperglycaemia (Wu et al., 2023). Currently, a rapidly growing of T2DM has become a health problem affecting nearly half a billion people worldwide (Magliano et al., 2021; Zhang et al., 2023). Therefore, it is necessary to develop effective drugs for the prevention and treatment of T2DM.

Bile acids (BAs) have a strong correlation with diabetes. The composition and level changes of BAs could be observed in patients with insulin resistance or T2DM (Du et al., 2022). Additionally, some studies have found that gastrointestinal homeostasis is jointly regulated by BAs and gut microbes, and the imbalance of this interaction may lead to the development of T2DM and other pathologies (Guo et al., 2022). As intestinal signaling molecules, BAs promote fibroblast growth factor-15 (FGF15, murine homologous gene of human FGF19), a gastrointestinal hormone, transmitting signals by activating the farnesoid X receptor (FXR), and play a vital role in glycolipid metabolism and energy metabolism (Meiring et al., 2022; Tawulie et al., 2023). Furthermore, cholesterol 7α hydroxylase (CYP7A1), as a rate-limiting enzyme in the classical BAs synthesis pathway, determines the rate of BAs synthesis and converts cholesterol to 7α-hydroxycholesterol (Chiang, 2013). Simultaneously, CYP7A1 activation is negatively regulated by FXR receptors. When FXR is activated in the liver or intestines, the expression of FGF15 increases, which in turn inhibits the expression of CYP7A1, thereby reducing the synthesis of BAs (Yan et al., 2022). Currently, synthetic FXR agonists such as obeticholic acid have been shown to be effective against insulin resistance and have been applied in clinical as a potential therapy for T2DM (Kuipers et al., 2014). Besides, FXR antagonists such as NDB and HS218 could effectively reduce blood glucose in obese mice and suppress the expression of gluconeogenic gene in db/db mice (Zhao et al., 2021). These researches suggest that FXR and BAs metabolism occupy a significative role in glycolipid metabolism.

Tibetan medicine is the most complete medical system in the ethnic minority medical system in China, with a history of more than 3,800 years. The unique medical theory and abundant medicinal plant resources on the Qinghai-Tibet Plateau provide favorable conditions for the treatment of diseases with Tibetan medicine (Zhao et al., 2021). In recent years, the study of Tibetan medicine in treating diabetes has been paid more and more attention. Some Tibetan herbs and preparations have been shown to be effective in treating T2DM and its complications, such as Tibetan medicine Lvluo, *Berberis kansuensis* Schneid, and Shibawei Hezi Liniao pills (Liu, 2018; Xu et al., 2021; Zheng et al., 2021). Ji-Ni-De-Xie (JNDX) is a hospital preparation prepared by the Tibetan Medicine Hospital in Dege County, Sichuan Province, China. It is comprised of *B. kansuensis* C.K.Schneid., *Curcuma longa* L., *Phyllanthus emblica* L., *Tribulus terrestris* L., *Carthamus tinctorius* L., *Terminalia chebula* Retz., *Astragalus floridus* Bunge, and *Adhatoda vasica* Nees (Table 1). It has been clinically employed in treating diabetes for over a decade, boasting a robust clinical foundation that ensures its safety and efficacy (Li et al., 2015; Luo et al., 2022a). Although the efficacy of JNDX in the treatment of T2DM is definite, its specific anti-diabetic mechanism remains unclear. Hence, to better understand the therapeutic effects and potential mechanisms of JNDX on T2DM, we established a T2DM rat model induced by a high-fat diet (HFD) combined with streptozotocin (STZ). In addition, targeted metabolomics based on high-performance liquid chromatography-triple quadrupole mass spectrometry (HPLC-QQQ-MS), metagenomics, real-time quantitative polymerase chain reaction (RT-qPCR), and Western blotting were combined to explore the potential mechanisms of JNDX in improving T2DM from the perspective of regulating BAs metabolism. The results of this study will provide important reference for the clinical application and drug development of JNDX.

**TABLE 1:** Medicinal information of Ji-Ni-De-Xie (JNDX).

Chinese name	Tibetan name (transliteration)	Latin name	Family name	Parts used
Xiao Bo Pi	སྐྱེར་ཤུན། (Jie Xing)	*Berberis kansuensis* C.K.Schneid.	Berberidaceae	Stem bark
Jiang Huang	ཡུང་བ། (Yong Wa)	*Curcuma longa* L.	Zingiberaceae	Rhizome
Yu Gan Zi	སྐྱུ་རུ་ར། (Ju Ru La)	*Phyllanthus emblica* L.	Euphorbiaceae	Fruit
Ji Li	གཟེ་མ། (Sai Ma)	*Tribulus terrestris* L.	Zygophyllaceae	Fruit
Hong Hua	གུར་གུམ། (Ku Kong)	*Carthamus tinctorius* L.	Compositae	Flower
He Zi	ཨ་རུ་ར། (A Ru La)	*Terminalia chebula* Retz.	Combretaceae	Fruit
Duo Hua Huang Qi	སྲད་སེར། (Sa Sai)	*Astragalus floridus* Bunge	Leguminosae	Herb
Ya Zui Hua	བ་ཤ་ཀ། (Ba Xia Ga)	*Adhatoda vasica* Nees	Acanthaceae	Branch and leaf

Note: The family name and Latin name from “The Plant List” Webservers (http://www. theplantlist.org).

## 2 Materials and methods

### 2.1 Materials

Ji-Ni-De-Xie was obtained from Dege County Tibetan Hospital (Institute of Tibetan Medicine) (Sichuan, China). Metformin hydrochloride tablets were purchased from Sino American Shanghai Shiguibao Pharmaceutical Co., Ltd (Shanghai, China) (Lot number: ABS9845); STZ (Lot number: WXBD7077V) was purchased from Sigma-Aldrich Co., St (Louis, United States). The enzyme-linked immunosorbent assay (ELISA) kits of insulin (Ins) (No. MM-0587R1), glycosylated serum protein (GSP) (No. MM-0735R1), tumor necrosis factor-α (TNF-α) (No. MM-0180R1), lipopolysaccharide (LPS) (No. MM-0647R1), glucagon-like peptide 1 (GLP-1) (No. MM-0033R2), and FGF15 (No. MM-0938R1) were obtained from Jiangsu Meimian Industrial Co., Ltd (Jiangsu, China). The ELISA kits of interleukin-6 (IL-6) (No. EK306/3-96) and IL-1β (No. EK301B/3-96) were provided by MULTISCIENCES (LIANKE) Biotech, Co., Ltd (Zhejiang, China). Triglyceride (TG) (No. C061-a), total cholesterol (TC) (No. C063-a), low-density lipoprotein cholesterol (LDL-C, No. C070-a), and high-density lipoprotein cholesterol (HDL-C) (No. C069-a) were purchased from Changchun Huili Biotech CO., LTD. Primary antibodies FXR/NR1H4 Rabbit mAb (1:1500, A24015, ABclonal Technology, China), Rabbit anti-CYP7A1 (1:1000, bs-21430R, Bioss, China), and anti-FGF15 (1:1000, sc-398338, Santa Cruz Biotechnology Inc., United States). Secondary antibodies goat anti-rabbit (1:10000, bs-0295G-HRP, Bioss, China) and HRP goat anti-mouse IgG (H + L) (1:10000, AS003, ABclonal Technology, China). β-actin (1:1000, GB15003, Servicebio, China).

### 2.2 Preparation and composition analysis of JNDX

JNDX was ground and sifted through No. 3 sieve, and then accurately weighed 0.1 g into 100 mL conical bottle. 50 mL 50% methanol was added into bottle, and then ultrasound was performed for 30min. After cooling, make up the weight with 50% methanol, and filter the supernatant with 0.22 μm microporous filter membrane.

The chemical constituents of JNDX were analyzed by HPLC-QQQ-MS (Agilent 1260, Agilent Technologies Inc., United States). The analysis was performed using a WondaSil C_18_-WR chromatographic column (4.6 × 250 mm, 5 μm). Then, the mobile phase was water containing 0.1% formic acid A)–methanol B). The column temperature was 25°C and the sample volume was 5.0 μL. The gradient program’s specific condition was set as follows: 0–1 min, 10%–45% B; 1–3 min, 45%–70% B; 3–4 min, 70%−70% B; 4–5 min, 70%–75% B; 5–7 min, 75%–75% B; 7–10 min, 75%–95% B; 10–20 min, 95% B. The mass spectrum conditions were as follows: electrospray ion source (ESI), high purity nitrogen (dry gas) as a collision gas, with a flow rate of 11 L∙min^-1^; capillary voltage was set at 4000 V (+) and 2500 V (−); temperature was maintained at 300°C with a pressure of 15 psi. Simultaneous determination of positive and negative ions was conducted in multiple reaction monitoring (MRM) mode.

### 2.3 Animals and treatment

Male Sprague-Dawley rats (weight 160–180 g, SPF level) and HFD were purchased from Chengdu Dashuo Experimental Animal Co., LTd. [Laboratory animal license number SCXK (Sichuan) 2020-030, Sichuan, China] and then adaptively housed in 25°C ± 2°C and relative humidity 50%–60% for 1 week. The rats were held in the Experimental Animal Center of Chengdu University of Traditional Chinese Medicine, and the water and diet were unrestricted supplied. The experimental unit license number is SYXK (Sichuan) 2020-124. Chengdu University of Traditional Chinese Medicine’s Animal Ethics Committee consented its approval to this research (the ethical approval number: 2018–15). The study was conducted in compliance with the guidelines set out by the National Health Institutes of China.

According to JNDX instruction, the adult clinical oral administration dose of JNDX is 1 to 2 pills/day (mass is 1.1537 g/pill). Thus, the maximum dose for adults is 0.0330 g/kg/d (assuming a body weight of 70 kg), and the high dose for rats in the present study is 0.330 g/kg/d, the medium dose is 0.165 g/kg/d, and the low dose is 0.083 g/kg/d (Chen, 2006). The specific groups and dosages are shown in Table 2. The JNDX powder was sieved through the 4^th^ screen, and 0.05% sodium carboxymethyl cellulose (CMC-Na) solvent was used to dissolve the powder and metformin (positive control group) respectively.

**TABLE 2:** Experimental dosages of rats *in vivo*.

Group	Volume of administration (mL/kg)	Dosage (g/kg/d)	concentration (g/mL)	Equivalent to multiple of clinical dosage
Normal	10.000	-	-	-
Model		-	-	-
Metformin		0.250	0.025	10.000
JNDX-H		0.330	0.330	10.000
JNDX-M		0.165	0.165	5.000
JNDX-L		0.083	0.083	2.500

### 2.4 Analysis of the chemical composition in rat serum after JNDX intervention by UPLC-Q-Exactive Orbitrap MS

After grinding, 50 g of JNDX was weighed and mixed with 10 times the volume of 50% ethanol. It was then heated and reflux-extracted twice for 2 h each time. The filtrate was mixed and concentrated under reduced pressure, resulting in a JNDX extract with a concentration of 0.625 g∙mL^-1^. Sprague-Dawley rats were randomly divided into normal group and JNDX group. The JNDX group received an oral gavage of 12.5 g∙kg^-1^ of JNDX extract, while the normal group received an equivalent volume of distilled water for three consecutive days. Oral gavage was administered twice daily, with the rats fasting but not restricted from water intake for 12 h before the final administration. Blood samples (0.5 mL) were collected from the orbital vein at 0.25 h, 0.5 h, 1 h, 1.5 h, 2 h, 3 h, 5 h, and 8 h after the final gavage, centrifuged at 3500 rpm for 10 min, and the serum was collected and stored at −80°C. For the JNDX group, 1 mL of serum was collected and mixed with 3 mL of acetonitrile to precipitate proteins. After centrifuging at 11000 rpm for 10 min, the supernatant was dried with nitrogen gas at 37°C. The residue was reconstituted with 200 μL of methanol, centrifuged at 11000 rpm for 10 min, and the supernatant was filtered through a 0.22 μm microporous membrane. Finally, we analyzed the filtrate using ultra high performance liquid chromatography with four stage rod/electrostatic field orbital trap high resolution mass spectrometry (UPLC-Q-Exactive Orbitrap MS) (Thermo-Fisher, United States).

The mass spectrometry conditions were as follows: using HESI ion source, positive ion detection mode with a spray voltage of +3500 V; negative ion detection mode with a spray voltage of −3000 V; sheath gas flow rate of 35.0 Arb, auxiliary gas flow rate of 10.0 Arb, and capillary temperature of 320°C. Full MS/dd-MS^2^ scan mode was employed with a Full MS resolution of 70,000 and dd-MS^2^ resolution of 17,500. The scan range was from m/z 100 to 1500.

High-performance liquid chromatography analysis was performed using a ZORBAX SB-C_18_ chromatographic column (150 mm × 2.1 mm, 1.8 μm). Then, the mobile phase was methanol A)–water containing 0.1% formic acid B). The column temperature was 30°C and the sample volume was 5.0 μL. The gradient program’s specific condition was set as follows: 0–20 min, 5%–20% A; 20–30 min, 50%–80% A; 30–35 min, 80%–95% A; 35–45 min, 95% A; 45–50 min, 95%–5% A (Luo et al., 2022a).

### 2.5 Induction of T2DM rats

Sprague-Dawley male rats were randomly divided into 2 groups, 8 were normal group, and the rest were model screening group for T2DM rats. The rats in the model group were fed with HFD for 4 weeks and then fasted for 12 h. On the following day, 1% STZ buffer solution (45 mg∙kg^-1^) was prepared for intraperitoneal injection in the model screening group. Besides, the rats in the normal group were injected with an equivalent amount of 0.1 mol∙L^-1^ citric acid-sodium citrate buffer solution (pH = 4.1). After STZ injection, the rats had free access to food and water, and fasting blood-glucose (FBG) was measured after fasting for 12 h at 72 h, 2 weeks, and 4 weeks, respectively. The FBG of each rat was measured three times by blood glucose meter (Anwen + code, Sinocare Inc.). The rats with three FBG indices greater than or equal to 11.0 mmol∙L^-1^ and the index of FBG steadily were considered as T2DM model rats.

A total of 40 model rats were selected and randomly divided into 5 groups: model group (model), metformin group (metformin), JNDX high-dose group (JNDX-H), JNDX medium-dose group (JNDX-M), and JNDX low-dose group (JNDX-L), with 8 rats in each group. All model rats were raised together with 8 rats in the normal group (normal). During the experiment, the normal group was fed with ordinary diet, and the model group and the JNDX group were fed with HFD.

### 2.6 Biochemical indicators assay

After the successful modeling of STZ in Sprague-Dawley rats, the rats were given continuous administration of JNDX for 40 days. Subsequently, the rats were fasted for 12 h and then FBG was measured by glucose meter. Blood samples were collected from abdominal aorta, and then the whole blood was left for 2 h, and centrifuged at 3500 rpm at 4°C for 15 min to obtain the serum. The serum was stored at − 80°C for detection. The contents of TG, TC, LDL-C, and HDL-C were measured by an automatic biochemical analyzer (BK-200, Shandong Biobase Scientific Instrument Co., LTD.). The ELISA kits were performed to detect the levels of GSP and Ins and then calculated the value of ho-meostasis model assessment of insulin resistance (HOMA-IR) and insulin sensitivity index (ISI). All experimental procedures were followed strictly with manufacturer’s instructions. HOMA-IR and ISI were calculated according to the following formulas:

$$\text{ISI}=\mathrm{Ln}\;\left[1/(FBG\;\left(mmol/mL\right)\times Ins\;\left(\mu U/mL\right)\right]$$

$$\text{HOMA}-\text{IR}=FBG\;\left(mmol/L\right)\times Ins\;\left(\mu U/mL\right)/22.5$$

(Xu et al., 2021).

### 2.7 Gut microbiota analysis

Fecal sample DNA of rats was extracted from Magnetic Soil and Stool DNA Kit (TIANGEN BIOTECH Co., LTD., Beijing, China) and metagenomic sequencing was performed. The DNA samples were fragmented into 350 bp fragments using ultrasonication for Illumina sequencing and subsequent PCR amplification. Following clustering, the library preparations were sequenced using the NovaSeq 6000 sequencing platform with PE150 read length.

The relative abundance information of gut microbiota was imported into R software (Version 4.2.2) for statistical analysis. The R package phyloseq was used to calculate α-diversity. α-diversity was studied to explore the complexity of species diversity using Shannon index (Lkhagva et al., 2021). Principal coordinates analysis (PCoA) based on Bray Curtis distance was conducted to explore the characteristics of gut microbiota. Anosim analysis was used to compare the similarities and differences in the composition of gut microbiota between the model and JNDX groups. Functional predictions of gut microbiota were performed using Kyoto Encyclopedia of Genes and Genomes (KEGG) and DIAMOND software (Kanehisa, et al., 2006; 2014; Buchfink, et al., 2015) based on metagenomic sequencing data. The Linear Discriminant Analysis Effect Size (LEfSe) software was employed to calculate the differences in functional pathways among groups at the family, genus, and species levels (LDA score >4.0, *p* < 0.05) (Segata, et al., 2011).

### 2.8 Effect of JNDX on BAs in T2DM rats based on HPLC-QQQ-MS analysis

The cryopreserved serum samples were thawed at room temperature and swirled evenly. Then, 100 μL serum sample was precisely measured and 10 μL internal standard (1.00 μg∙mL^-1^) (Cholic acid-d_4_, Lot number: ISO-13098, Nanjing Beiyu Biotechnology Co., LTD.) and 200 μL chromatograph-grade acetonitrile were added. The mixture swirled at room temperature for 2 min, stood for 10 min, and centrifuged at 11 000 rpm at 4°C for 10 min. The supernatant was processed through 0.22 μm microporous filter membrane. The serum samples of rats in each group were analyzed by a triple quadrupole mass spectrometer (Agilent G6420A, Agilent Technologies Inc., United States). The analysis method was established on the basis of our previous research work (Yi, 2022).

### 2.9 Enzyme-linked immunosorbent assay

The levels of LPS, IL-6, IL-1β, TNF-α, GLP-1, and FGF15 were estimated using ELISA kits. The experiment process was carried out in strict accordance with the manufacturer’s instructions.

### 2.10 RNA isolation and real-time quantitative polymerase chain reaction analysis

100 mg of the tissue was taken and fully grinded in 1 mL of pre-cooled lysis buffer. Total RNA was extracted from tissue using Trizol (Lot number: 9109, TAKARA). The concentration of total RNA was measured by ultramicro quantitative nucleic acid protein detector (EVA 3100, Monad Biotechnology Co. LTD., Jiangsu, China). Subsequently, 1 μg RNA template was prepared for reverse transcription using a M-MLV Reverse Transcriptase kit (M170A, Promega Biotech Co., Ltd., Beijing, China) and iScript ™ cDNA Synthesis Kit (1708890, Bio-Rad Laboratories, Inc., United States) was used to synthesize cDNA. Finally, RT-qPCR was performed via fluorescent quantitative PCR instrument (CFX Connect, Bio-Rad Laboratories, Inc., United States). The total reaction system of fluorescence quantitative detection was 10 μL. In addition, the amplification steps were as following: 95°C, 5 min for initial denaturation step, and 95°C, 10 s to 60°C, 30 s for 40 cycles of denaturation, and anneal at 65°C–95°C, 0.5°C/5 s. The primer sequences in this study were presented in Table 3. The 96-well plate was put into the fluorescence quantitative PCR instrument, and the detection was set on the Bio-Rad CFX Manager. The expression multiple was calculated using the ΔΔCT method, and the differences in the samples of relative mRNA expression were normalization by reference genes (GAPDH): ΔCT = CT target gene − CT reference gene, ΔΔCT = ΔCT treatment sample − ΔCT control sample, the analyzed formula = 2^−ΔΔCT^ (Su et al., 2023).

**TABLE 3:** Real-time quantitative polymerase chain reaction primer sequences used in this study.

Gene	Forward primers (5′–3′)	Reverse primers (5′–3′)
FXR	CAT​CAT​GGC​TTC​CGG​TTC​AG	TCC​CCA​CCT​TCC​TTT​CCA​TC
FGF15	AAG​TGG​AGT​GGG​CGT​ATT​GT	AGT​GGA​CCT​TCA​TCC​GAC​AC
GAPDH	AAG​TTC​AAC​GGC​ACA​GTC​AA	TCT​CGC​TCC​TGG​AAG​ATG​G

### 2.11 Western blot analysis

Animal tissues were homogenized and lysed using freezing grinder (JXFSTPRP-CL, Shanghai Jingxin Industrial Development Co., Ltd., Shanghai, China) and RIPA lysis buffer (AR0102S, BOSTER, China). Then, the samples were centrifuged at 10,000 g for 10 min at 4°C. The supernatant of each sample was obtained and the protein concentration of the sample was measured with BCA kit (AR0146, BOSTER, China). Whereafter, the SDS-PAGE sample loading buffer (AR1112-10, BOSTER, China) was added in total protein and denatured at 100°C for 10 min to obtain WB samples. 10% PAGE high resolution color gel was prepared to separate proteins of different molecular weight, and the isolated proteins were transferred to polyvinylidene difluoride (PVDF) membranes, which were then blocked with 5% BSA at room temperature for 90 min. The membranes were placed in incubator box containing primary antibody at 4°C overnight: FXR (1:1500), CYP7A1 (1:1000), and FGF15 (1:1000). On the second day, after the recovery of the primary antibody, membranes were washed three times with TBST. Next, membranes covered with the second antibody (1:10000) and incubated for 90 min at room temperature. After incubation, the membranes were washed three times and exposed to ultra-sensitive ECL chemiluminescent substrate kit (BL520A, Biosharp, China). Finally, visualizer (ChampChemi 610 Plus, Beijing Sage Creation, China) was employed to visualize the protein bands and ImageJ software (version 1.37V, NIH, United States) was performed to quantify the relative gray value of bands.

### 2.12 Statistical analyses

The data were presented as means ± standard deviation (SD). GraphPad Prism 8 (San Diego, CA, United States) was performed for the statistical analysis. One-way analysis of variance (ANOVA) was conducted to determine the *p*-value, with statistical significance established at a *p*-value <0.05.

## 3 Results

### 3.1 HPLC-QQQ-MS analysis of chemical constituents in JNDX

The chemical constituents of JNDX were analyzed by HPLC-QQQ-MS technique. A total of 12 constituents (magnoflorine, berberine, curcumin, jateorrhizine, demethoxycurcumin, gallic acid, chebulagic acid, ferulic acid 4-O-β-D-glucopyranoside, hydroxysafflor yellow A, rutin, ferulic acid, and ellagic acid) were identified in JNDX by comparing their retention time and mass spectrum information with standard compounds. In addition, the contents of the 12 constituents in JNDX were calculated according to their calibration curves established by the corresponding standard compounds. The results showed that gallic acid had the highest content (18.11 mg∙g^-1^), followed by chebulagic acid (4.98 mg∙g^-1^), magnoflorine (3.27 mg∙g^-1^), ellagic acid (3.17 mg∙g^-1^), ferulic acid 4-O-β-D-glucopyranoside (3.07 mg∙g^-1^), and ferulic acid (1.83 mg∙g^-1^). The content determination results, mass spectrometry information and chromatogram of JNDX are shown in Supplementary Table S1, S2 and Supplementary Figure S1.

### 3.2 UPLC-Q-Exactive Orbitrap MS analysis of chemical compounds in rat serum after JNDX intervention

A total of 45 chemical compounds in rat serum after JNDX intervention were identified using UPLC-Q-Exactive Orbitrap MS technology, including 22 prototype components and 23 metabolites. The detailed chemical compounds in rat serum and the diagram of positive and negative ion flow are shown in Supplementary Figure S2 and Supplementary Table S3. These components included berberine, magnoflorine, ferulic acid, ellagic acid, jateorrhizine, demethoxycurcumin, and curcumin. They mainly originated from the combination of *B. kansuensis* C.K.Schneid., *C. longa* L., *P. emblica* L., and *T. chebula* Retz., suggesting that these four herbs contributed significantly to the treatment of T2DM with JNDX. In conclusion, the results indicate that berberine, magnoflorine, ferulic acid, ellagic acid, jateorrhizine, demethoxycurcumin, and curcumin may be the effective components of JNDX treating T2DM.

### 3.3 JNDX reduced blood glucose, ameliorate insulin resistance, and insulin sensitivity in T2DM rats

**FIGURE 1:**
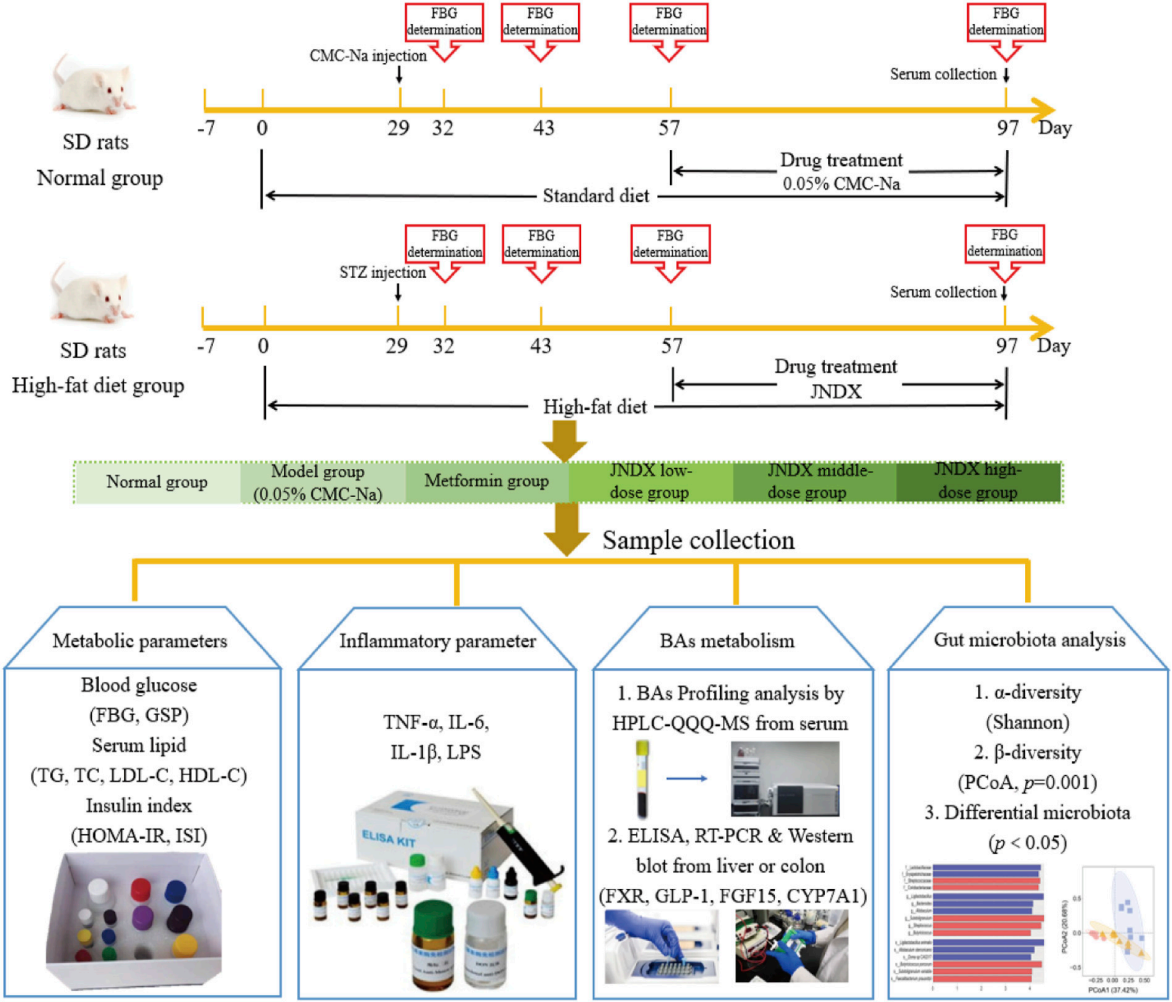
The specific experimental procedure in this study.

**FIGURE 2:**
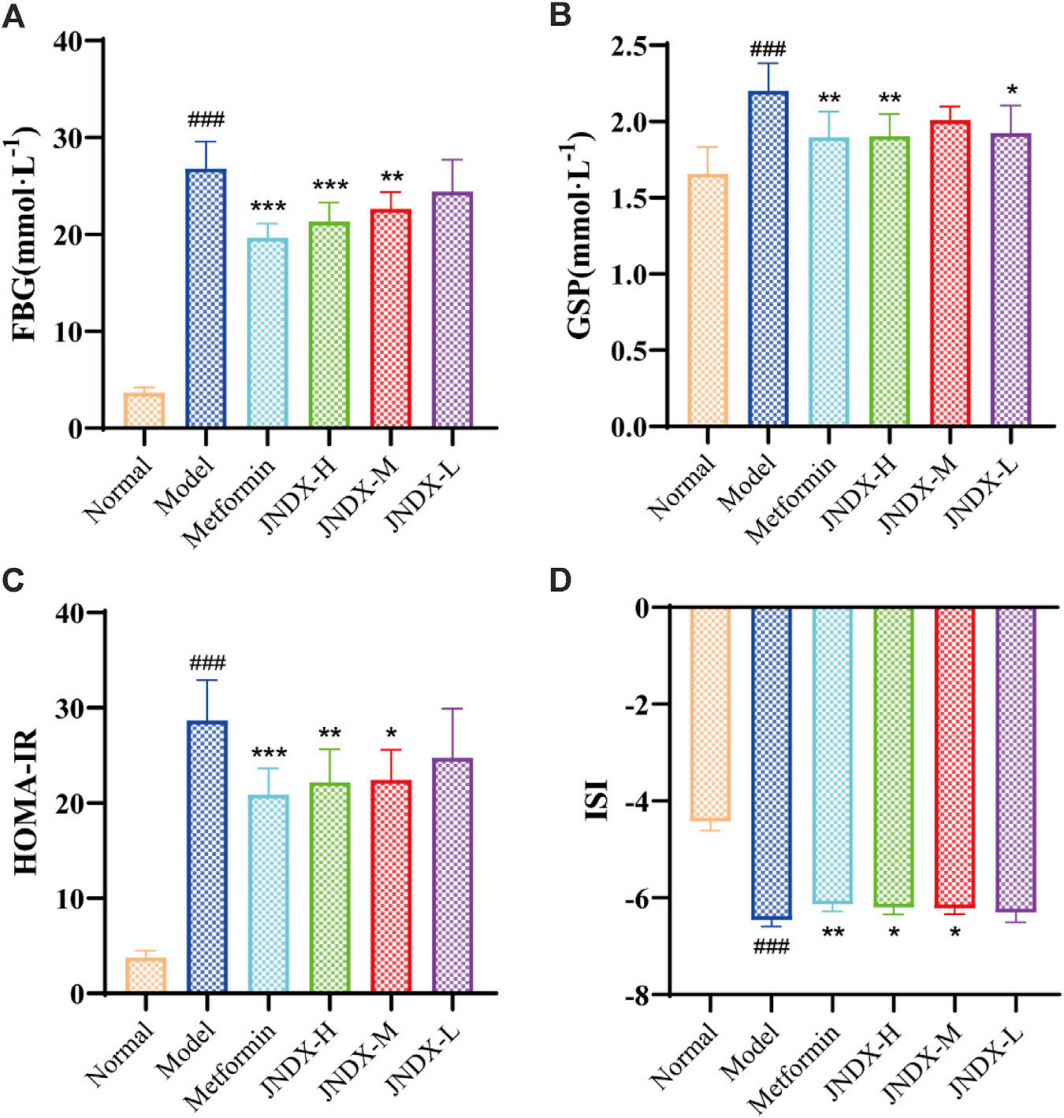
Effects of JNDX on FBG **(A)**, GSP **(B)**, HOMA-IR **(C)**, and ISI **(D)**. All data were represented as mean ± SD. ^#^ *p* < 0.05, ^##^ *p* < 0.01, ^###^ *p* < 0.001 vs the Normal group, *p < 0.05, **p < 0.01, ***p < 0.001 vs the Model group.

After the successful modeling of T2DM rats, the typical diabetes symptoms of eating more, drinking more water, excreting more, while the weight loss were obvious. Figure 1 depicts the specific experimental procedure. The effect of JNDX on T2DM rats was evaluated by measuring relevant blood glucose and insulin-related indices. Figure 2A shows that FBG is significantly higher in model group than in normal group (*p* ˂ 0.001). After 40 days of administration, both the high dose and medium dose groups of JNDX could significantly reduce FBG (*p* ˂ 0.01), while the effect of FBG reduction was not significantly different in the low dose group. These results indicate that JNDX could significantly reduce FBG in T2DM rats. In addition, GSP was similar to glycosylated hemoglobin and reflected the average blood glucose concentration over the past 1–3 weeks. The results indicate that the GSP level of the T2DM rats in the model group is significantly higher than that in the normal group (*p* ˂ 0.001). After 40 days of administration, GSP content was obviously decreased at JNDX-H and JNDX-L group compared with model group (*p* ˂ 0.05) (Figure 2B).

Ins value of rats in each group were measured by kit. HOMA-IR and ISI were calculated according to FBG and ins of rats after the last administration. Results are shown in Figures 2C, D. Compared with the normal group, the HOMA-IR of the model group was markedly increased (*p* ˂ 0.001), and the ISI was significantly decreased (*p* ˂ 0.001). Compared with the model group, HOMA-IR was obviously decreased (*p* ˂ 0.05), while the ISI was increased observably (*p* ˂ 0.05) in JNDX-H and JNDX-M groups. The results suggest that JNDX can improve insulin resistance and insulin sensitivity in T2DM rats.

### 3.4 Effects of JNDX on LPS and inflammatory cytokines in T2DM rats

**FIGURE 3:**
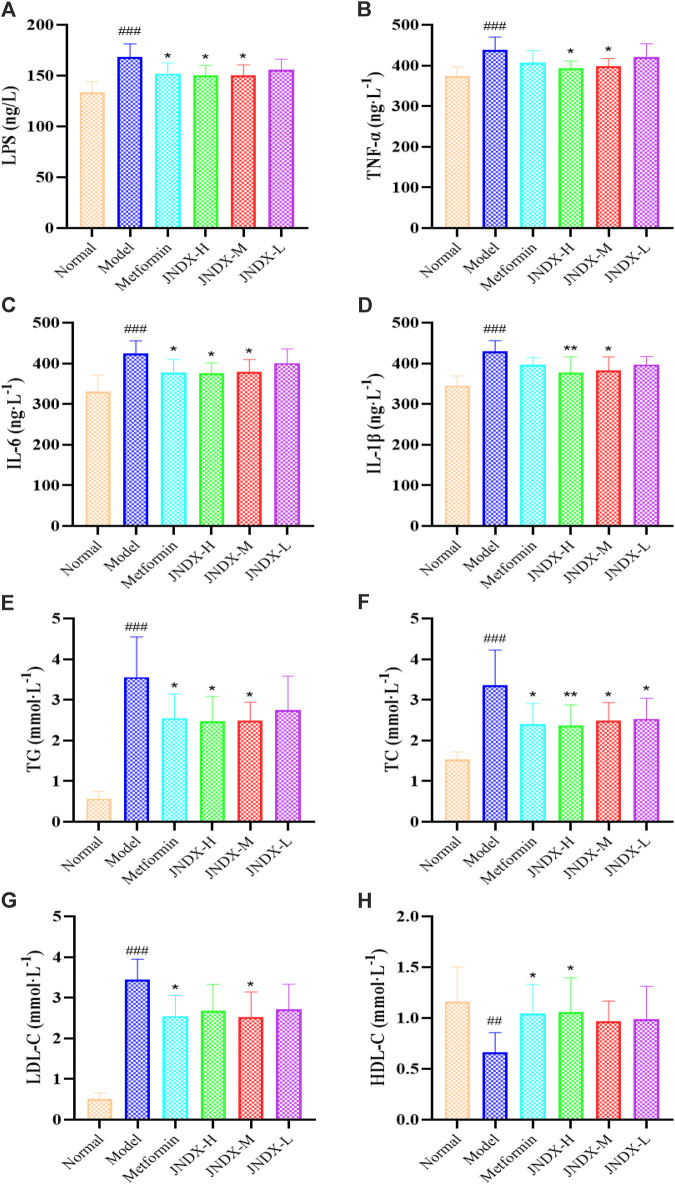
Effects of JNDX on LPS **(A)**, pro-inflammatory cytokines TNF-α **(B)**, IL-6 **(C)**, and IL-1β **(D)**, and lipid indices TG **(E)**, TC **(F)**, LDL-C **(G)**, and HDL-C **(H)**. All data were represented as mean ± SD. ^#^ *p* < 0.05, ^##^ *p* < 0.01, ^###^ *p* < 0.001 vs the Normal group, *p < 0.05, **p < 0.01, ***p < 0.001 vs the Model group.

To further investigate the effect of JNDX on inflammation in T2DM rats. We found that the serum levels of LPS, IL-1β, TNF-α, and IL-6 were significantly increased in model group (Figures 3A–D). Both high- and medium-dose JNDX markedly decreased the level of LPS, IL-1β, TNF-α, and IL-6 (*p* ˂ 0.05), whereas the low-dose JNDX decrease mildly in these indexes. These results demonstrate that JNDX can improve inflammation in T2DM rats.

### 3.5 Effects of JNDX on blood lipids (TG, TC, LDL-C and HDL-C) in T2DM rats

Through the detection of blood lipid indexes of rats in each group, we found that TG, TC, and LDL-C of T2DM rats in model group were upregulated compared with normal rats (*p* ˂ 0.001). Nevertheless, after JNDX intervention, these indicator levels were reversed (Figures 3E–G). Besides, the expression of HDL-C was lower in the model group than in the normal group, and JNDX-H could noteworthily increase the level of HDL-C (*p* ˂ 0.05) (Figure 3H). These results suggest that JNDX can ameliorate the blood lipid in T2DM rats.

### 3.6 Effects of JNDX on gut microbiota in T2DM rats

**FIGURE 4:**
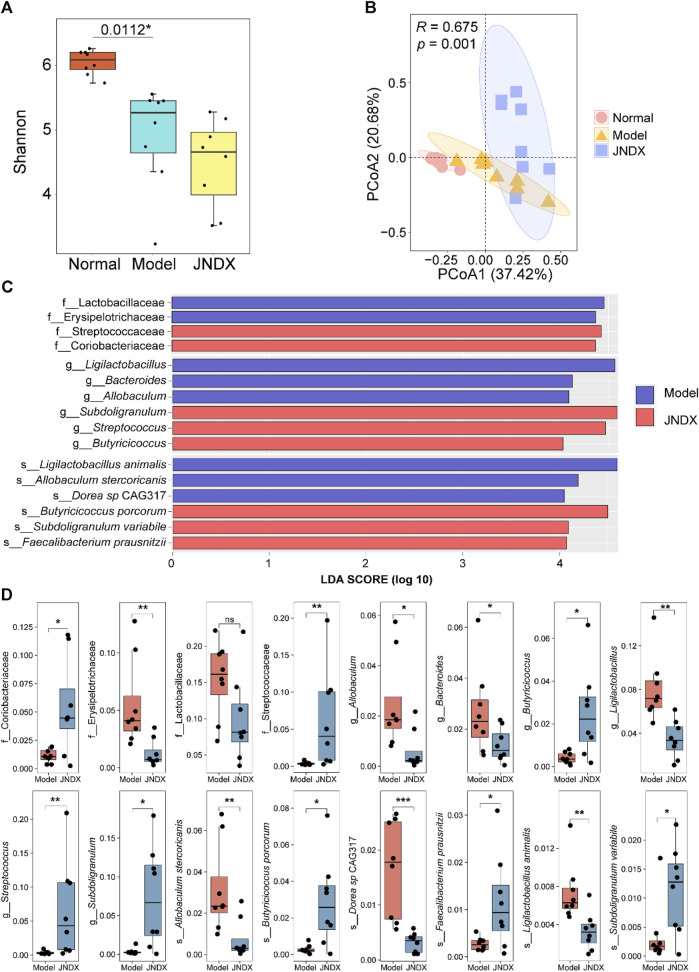
Effects of JNDX on gut microbiota. **(A)** Differences in gut bacterial diversity among the 3 groups of rats based on Shannon index. **(B)** Principal coordinates analysis (PCoA) analysis based on Bray Curtis distance was performed to explore the gut microbiota characteristics between the 3 groups. *R* > 0 indicates that the between-group differences were greater than the within-group differences. Reliability of statistical analyses was expressed as *p*-value. **(C)** LEfSe analysis showed significant enrichment of gut microbiota in the model and JNDX groups at the family, genus, and species levels. Taxa with LDA scores >4 are shown. **(D)** Kruskal Wallis variance analysis showed the differential expression of gut microbiota between the model group and the JNDX group.

Metagenomic analysis of fecal samples was performed to assess the effect of JNDX on gut microbiota in T2DM rats. The Shannon index was significantly different between the normal and model groups (*p* = 0.0112). There was no significant difference between the model and the JNDX group (Figure 4A). The results showed that JNDX had little effect on the α diversity of gut microbiota. However, PCoA analysis showed a clear separation among the three groups, suggesting that JNDX significantly affected the composition of the gut microbiota (PCoA1, 37.42%, PCoA2, 20.68%, *p* = 0.001) (Figure 4B). The relative abundance of gut microbiota in normal, model, and JNDX groups at the family, genus, and species levels are presented in Supplementary Figure S3 and Supplementary Table S4. Figures 4C, D show significant enrichment of gut microbiota in model and JNDX rats at the family, genus, and species levels based on LEfSe analysis. The results suggest that JNDX can improve the gut microbiota disorder in T2DM rats by increasing the relative abundance of some bacteria (Coriobacteriaceae, Streptococcaceae, *Butyricicoccus*, *Streptococcus*, *Subdoligranulum*, *Butyricicoccus porcorum*, *Faecalibacterium prausnitzii*, and *Subdoligranulum variabile*) and decreasing the relative abundance of some bacteria (Erysipelotrichaceae, Lactobacillaceae, *Allobaculum*, *Bacteroides*, *Ligilactobacillus*, *Allobaculum stercoricanis*, *Dorea sp* CAG317, and *Ligilactobacillus animalis*). Among them, *Butyricicoccus*, *B. porcorum*, and *F. prausnitzii* have been proven to be beneficial bacteria, promoting improvement in T2DM (Heinken et al., 2014; Xu et al., 2015). Additionally, *Allobaculum* and *Bacteroides* have been demonstrated to be harmful bacteria, detrimental to the treatment of T2DM (Wang et al., 2020; Chen et al., 2023). Notably, some bacteria such as *Streptococcus* and *Bacteroides* are closely associated with BAs metabolism, indicating that JNDX may further influence BAs profile in T2DM rats.

### 3.7 Effects of JNDX on bile acids metabolism in T2DM rats

**FIGURE 5:**
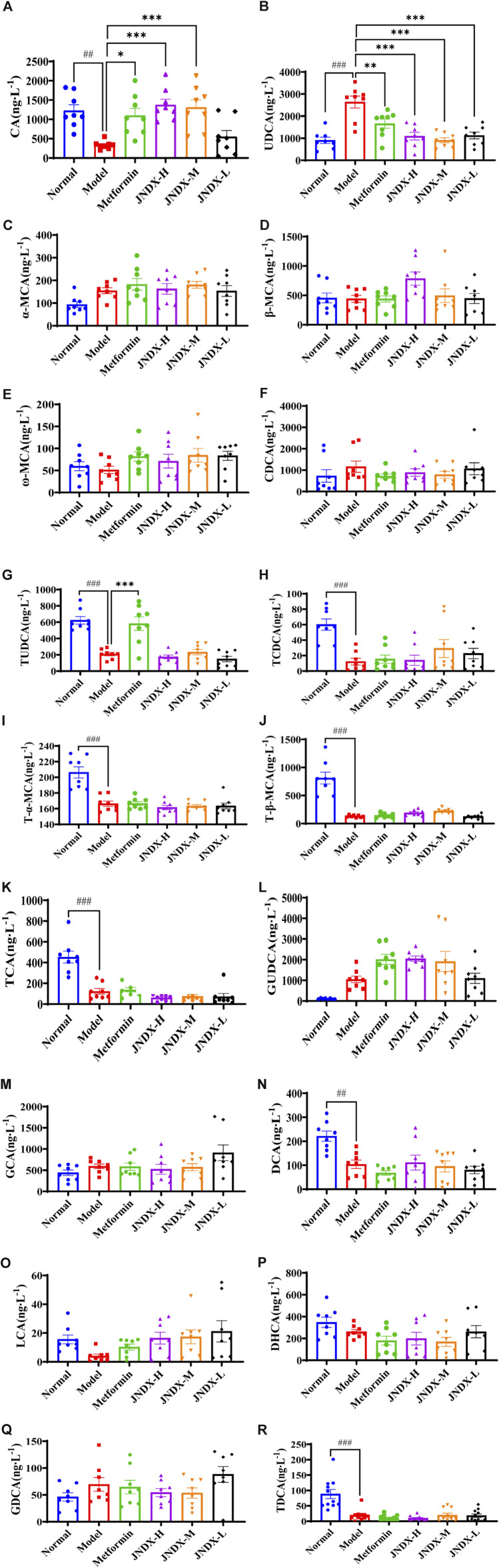
Effects of JNDX on serum BAs in T2DM rats. Comparison of differences in 18 serum BAs among different groups of rats **(A)** CA, **(B)** UDCA, **(C)** α-MCA, **(D)** β-MCA, **(E)** ω-MCA, **(F)** CDCA, **(G)** TUDCA, **(H)** TCDCA, **(I)** T-α-MCA, **(J)** T-β-MCA, **(K)** TCA, **(L)** GUDCA, **(M)** GCA, **(N)** DCA, **(O)** LCA, **(P)** DHCA, **(Q)** GDCA, **(R)** TDCA, all data were represented as mean ± SD. ^#^ *p* < 0.05, ^##^ *p* < 0.01, ^###^ *p* < 0.001 vs the normal group, *p < 0.05, **p < 0.01, ***p < 0.001 vs the model group.

HPLC-QQQ-MS analyzed 18 BAs, including cholic acid (CA), dihydrocaffeic acid (DHCA), glycocholic acid (GCA), and glycoursodeoxycholic acid (GUDCA), *etc.* in the serum of rats. The data of each group were analyzed by GraphPad Prism software, and the results are shown in Figure 5. The total BAs, total primary BAs, and total secondary BAs are illustrated in Supplementary Figure S4. Compared to the normal group, the contents of 6 primary BAs [CA, tauroursodeoxycholic acid (TUDCA), taurochenodeoxycholic acid (TCDCA), tauro-alpha-muricholic acid (T-α-MCA), tauro-beta-muricholic acid (T-β-MCA), and taurocholic acid (TCA)] in the model group were significantly decreased (*p* ˂ 0.05), and the secondary BAs such as deoxycholic acid (DCA) and taurodeoxycholic acid (TDCA) were also markedly reduced (*p* ˂ 0.001) compared to the normal group. Besides, the primary BAs ursodeoxycholic acid (UDCA) was significantly increased (*p* ˂ 0.001) and CA was remarkable decreased (*p* ˂ 0.01) in comparison to those in the normal group. Nevertheless, compared with the model group, UDCA level was significantly decreased (*p* ˂ 0.001) and CA level was significantly increased (*p* ˂ 0.001) observably in JNDX group. The results indicate that JNDX can affect BAs metabolism.

### 3.8 Effects of JNDX on the FXR-FGF15 signaling pathway in T2DM model rats

#### 3.8.1 Effect of JNDX on serum FGF15 and GLP-1 in T2DM rats

**FIGURE 6:**
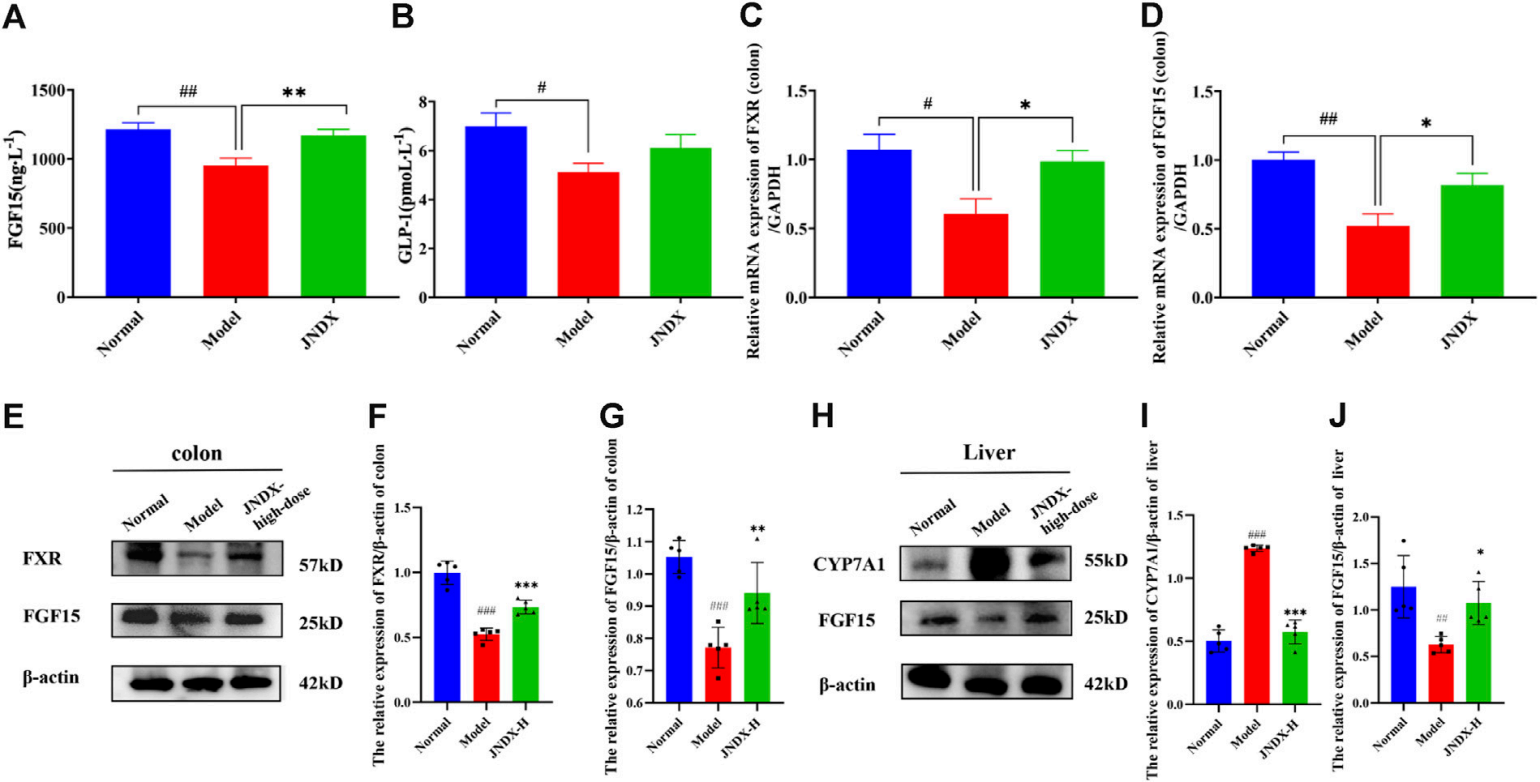
Effects of JNDX on serum FGF15 **(A)** and GLP-1 **(B)** in T2DM rats. T2DM rats were treated with JNDX to observe its impacts on the relative mRNA expression of FXR **(C)** and FGF15 **(D)**, as well as FXR and FGF15 protein levels in the colon **(E–G)**, and CYP7A1 and FGF15 protein levels in the liver **(H–J)**. All data were represented as mean ± SD. ^#^ *p* < 0.05, ^##^ *p* < 0.01, ^###^ *p* < 0.001 vs the Normal group, *p < 0.05, **p < 0.01, ***p < 0.001 vs the Model group.

The results showed that FGF15 and GLP-1 in model group were declined significantly (*p* ˂ 0.05) compared with normal group. Besides, compared with model group, FGF15 was distinctly risen after 40 days of administration of JNDX (*p* ˂ 0.01) (Figure 6A). Although there was no markedly difference in serum GLP-1 among all groups, it had some improvement effect as well (Figure 6B). The results reflect that JNDX can increase the expression of FGF15 in T2DM rats.

#### 3.8.2 Effect of JNDX on FXR and FGF15 mRNA expression in the intestine of T2DM rats

The expression of related mRNA (FXR, FGF15) in the FXR/FGF15 pathway was detected by RT-qPCR. we found that the mRNA levels of FXR and FGF15 in the colon of the model group were significantly reduced (*p* ˂ 0.05) compared with the normal group. After 40 days of JNDX intervention, the levels of FXR and FGF15 were upregulated (*p* ˂ 0.05) (Figures 6C, D). The results show that JNDX can increase the relative mRNA expression of FXR and FGF15 in intestinal with T2DM rats.

#### 3.8.3 Effect of JNDX on FXR/FGF15 pathway related protein expression in intestine and liver of T2DM rats

FXR was a key receptor for BAs. It was highly expressed in the liver and intestines. Notably, activating intestinal FXR, which was once speculated to be a helpful therapy, treats metabolic diseases. In our study, we detected FXR and FGF15 proteins in rat′s colon and CYP7A1 and FGF15 proteins in rat′s liver in the FXR/FGF15 pathway. The results showed that in the model group, the levels of FXR and FGF15 in T2DM rat were remarkable reduction, whereas the expression of CYP7A1 was observably increased compared with the normal group (*p* ˂ 0.001). After intervening of high dose JNDX, the levels of FXR and FGF15 were increased (*p* ˂ 0.01) (Figures 6E–G, J) and CYP7A1 was downregulated in comparison to those in the model group (*p* ˂ 0.001) (Figures 6H, I). These results reveal that JNDX ameliorates the symptoms of T2DM rats via regulating the FXR/FGF15 signal pathway.

## 4 Discussion

In the present study, we successfully established a T2DM rat model by combining HFD and STZ (45 mg/kg), and the results showed that the T2DM model group had obvious characteristics of higher blood lipids and blood glucose compared to normal group. In addition, the elevated levels of FBG, GSP, HOMA-IR, ISI, TG, TC, LDL-C, LPS, TNF-α, IL-1β, and IL-6 indicated increased insulin resistance, decreased insulin sensitivity, and increased inflammation in T2DM rats. Furthermore, the BAs profile and gut microbiota of T2DM rats were significantly disturbed. We subsequently found a decrease in the protein and mRNA expressions of FXR (colon) and FGF15 (colon, liver), while the protein level of CYP7A1 (liver) increased in T2DM rats. Interestingly, these indicators were reversed after taking JNDX.

T2DM is a chronic condition that affects the way your body processes blood glucose. Insulin is a hormone produced by the pancreas that helps glucose from food enter the body’s cells to be used for energy. When people have T2DM, their bodies either resist the effects of insulin or do not produce enough insulin to maintain normal glucose levels. As a result, glucose accumulates in the bloodstream instead of being used for energy, leading to high blood sugar levels (Al-kuraishy et al., 2023). The general treatment for T2DM may include oral medications, insulin therapy, and lifestyle modifications. Metformin, tolbutamide and GLP-1 agonists (e.g., exenatide and repaglinide) are employed in reducing the hyperglycemia at present (Uppal et al., 2018). Nevertheless, these drugs will produce some adverse reactions, such as vomiting, headache, and diarrhea (Doyle and Egan, 2007). Traditional herbal medicine has been used to treat diabetes for many years, and the advantage of it lies in its comprehensive use of various ingredients and effects of herbs, focusing on the overall balance and individual treatment. Importantly, traditional herbs are highly regarded for their low side effects and good efficacy, making them a preferred choice for treating diabetes compared to other anti-diabetic drugs (Li et al., 2021). Hence, it is meaningful to study the anti-diabetic effect and mechanism of traditional Tibetan medicine.

In recent years, research on Tibetan medicine for the treatment of diabetes has gradually received attention. Diabetes is a modern medical term, which in Tibetan medical theory, can be classified as a “Jing Ni Sa Ku” (གཅིན་པ་མངར་འགྱུར་གྱི་ནད་) disease. The diagnosis of T2DM in the “Four Medical Classics” divides diabetes into three major types and a total of 20 subtypes based on the color, odor, and nature of urine (four types of Long རླུང་།, six types of Chiba མཁྲིས་པ།, and ten types of Peigen བད་ཀན།) (Zhong, 2014). Currently, some Tibetan medicinal materials and compound preparations have been studied and used for the prevention and treatment of T2DM and its complications, such as JNDX. As a hospital preparation, the clinical function of JNDX has been shown to be effective. Moreover, in our previous research, we investigated the chemical composition and quality criteria of JNDX, and predicted its therapeutic target for T2DM through network pharmacology (Peng et al., 2022; Luo et al., 2022a; Li et al., 2015). However, the possible mechanisms of JNDX treating T2DM have not yet been explored. Hence, we established a T2DM rat model to investigate the underlying mechanisms of JNDX *in vivo*.

The commonly used method for modeling T2DM in male rats involves inducing insulin resistance through a HFD and subsequently impairing pancreatic β-cell function by injecting a small dose of STZ (Skovsø, 2014). This model has been noted for its effective and economic advantages (Ghasemi et al., 2014). In this study, the T2DM rats consistently showed FBG levels exceeding 11.0 mmol/L, along with typical diabetic symptoms such as polyphagia, polydipsia, and polyuria throughout the modeling period. Furthermore, many clinical studies have observed elevated levels of inflammatory mediators in the blood circulation of T2DM patients (Huang et al., 2022). Therefore, T2DM is closely associated with hyperglycemia, hyperlipidemia, and inflammation. We detected the levels of FBG, GSP, HOMA-IR, ISI, TG, TC, LDL-C, HDL-C, LPS, TNF-α, IL-1β, and IL-6 to evaluate the anti-diabetic effect of JNDX. Among them, the increasing of LPS could bind with leukocytes in the bloodstream, thereby inducing the expression of pro-inflammatory cytokines TNF-α, IL-1β, and IL-6 and resulting in inflammatory response (Guan et al., 2023). Furthermore, impaired pancreatic function in diabetic patients often leads to abnormal lipid metabolism, so evaluating lipid parameters (TG, TC, LDL-C, and HDL-C) in T2DM patients has clinical value (Bai et al., 2021). Studies have indicated that the levels of TG, TC, and LDL-C in diabetic patients are higher than those in non-diabetic individuals, while the level of HDL-C is lower (Zhou et al., 2023). Therefore, reducing blood glucose, blood lipid levels, and inflammation is a beneficial approach to improving T2DM.

In the present study, gut microbiota dysbiosis is significantly improved in T2DM rats after 40 days of JNDX administration, including increased relative abundance of *Butyricicoccus* and *F. prausnitzii*, and decreased relative abundance of *Allobaculum* and *Bacteroides*. Among them, *F. prausnitzii* is a well-known bacterium that produces butyrate, and the production of butyrate contributes to maintaining gut health (Heinken et al., 2014). Besides, a study has found the similar results that some traditional Chinese herbal formulas rich in *F. prausnitzii* have the potential to alleviate T2DM (Xu et al., 2015). Moreover, *Butyricicoccus* is also a bacterium that produces butyrate, which has a positive effect on improving T2DM. Furthermore, in the regulation of the gut microbiota, an increase in certain harmful bacteria could aggravate gut microbiota dysbiosis. *Bacteroides* enterotype is an independent risk factor for T2DM, which attributes to elevated LPS levels leading to diminished insulin sensitivity (Wang et al., 2020). *Allobaculum* has been shown to produce trimethylamine oxide, which can not only promote fat production by inhibiting the BA-mediated hepatic FXR signaling, but also induce insulin resistance, thus impacting blood glucose homeostasis and the occurrence and development of T2DM (Chen et al., 2023). Furthermore, *Streptococcus thermophilus* MN-ZLW-002 regulates gut microbiota by adjusting the composition of BAs and reduces the levels of high lipid and glucose. (Luo et al., 2022b).

BAs have been proven to be closely associated with T2DM. Our study demonstrates that JNDX can improve BAs disorder in T2DM rats. In addition, BAs can ameliorate the T2DM symptoms by relieving inflammatory response, promoting insulin secretion, relieving endoplasmic reticulum stress, and inhibiting insulin resistance (Staley et al., 2017; Maghsoodi et al., 2019). BAs are classified into primary and secondary BAs based on their origins. CA and chenodeoxycholic acid (CDCA), the primary BAs, are general synthesized in the human liver. It is noteworthy that bile salt hydrolase (BSH) enzymatically converts primary conjugated BAs into unconjugated BAs. Subsequently, through epimerization, UDCA is generated, followed by the formation of secondary BAs through hydroxylation catalyzed by 7α-hydroxylase. Secondary BAs such as DCA and lithocholic acid (LCA) are produced in the human body. Nevertheless, in rodents, secondary BAs can be generated including DCA and LCA (Xiang et al., 2021). Primary and secondary BAs together constitute the bile acid spectrum (Prawitt et al., 2011; Zhao et al., 2020). Many clinical and animal experiments have found changes in the BAs spectrum of T2DM patients and animals. The investigation demonstrates that the T2DM group showed significant changes in the content of primary BAs (e.g., CA, UDCA, and T-α-MCA) and secondary BAs (e.g., DCA, TUDCA, and TDCA), compared to the normal group (Meier, 2012; Fang et al., 2015; Wahlström et al., 2016). Additionally, CA and CDCA increase the expression of FXR receptors in liver tissue, promoting the secretion of GLP-1, which beneficially impacts glucose metabolism. By contrast, studies have noted that increased levels of most conjugated BAs (GCA, GCDCA, TCA, and TCDCA) are associated with an increased risk of T2DM (Lu et al., 2021). Some studies have indicated that CA is an agonist of FXR, while UDCA acts as an antagonist of FXR (Du et al., 2022; Li et al., 2024). Accordingly, our research findings suggest that the elevation of CA and reduction of UDCA levels in circulating serum activate FXR expression, thereby modulating the FXR/FGF15 pathway, affecting BAs synthesis, and improving T2DM.

**FIGURE 7:**
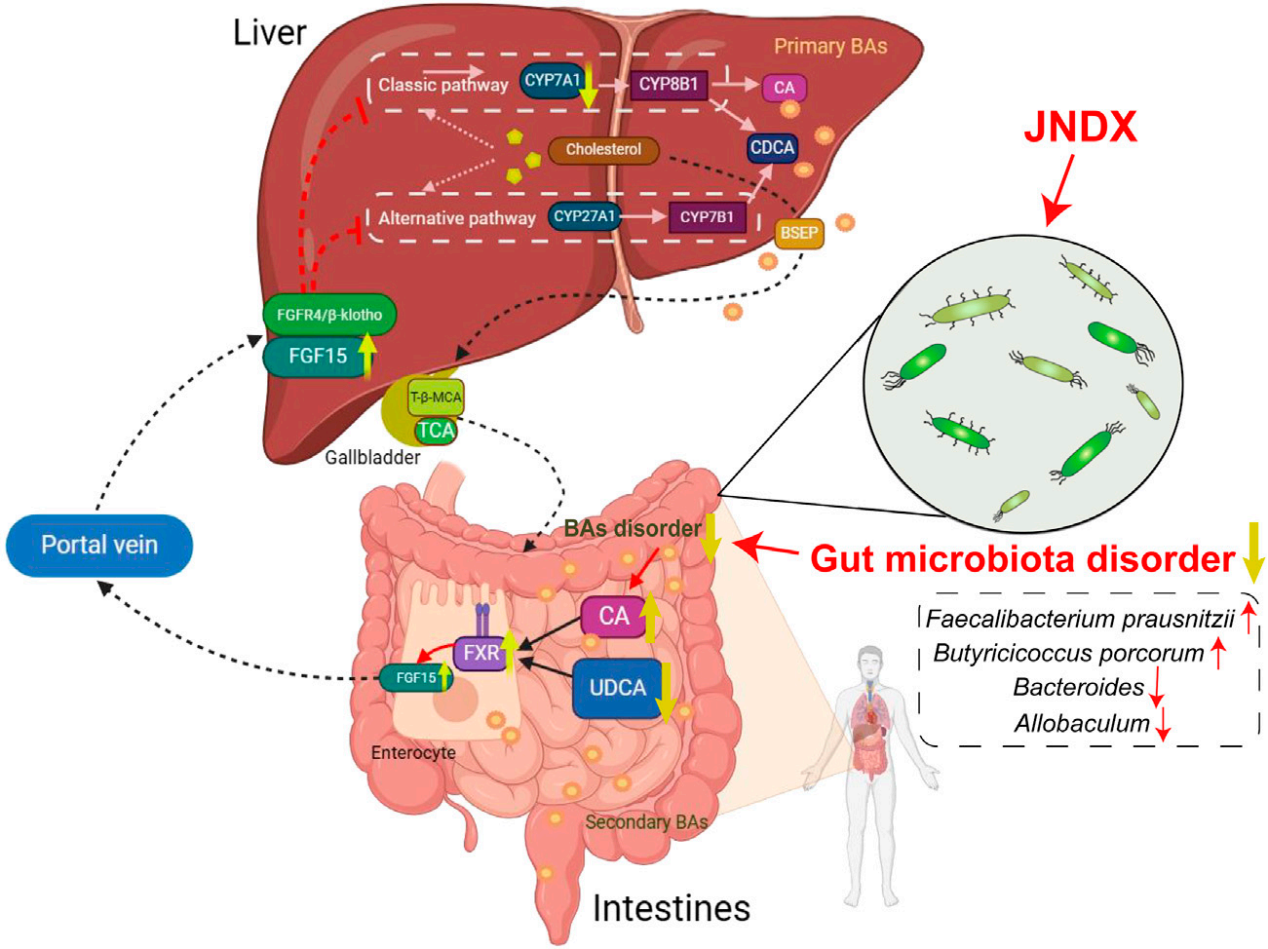
An overview diagram of the impact of JNDX on gut-liver axis and FXR/FGF15 signaling. 1) In the intestine: JNDX improves gut microbiota and bile acids metabolism (e.g., CA and UDCA) disorders, and then activates FXR, thereby promoting the production of FGF15. 2) In hepatocytes: FGF15 binds to the liver FGF4/βKlotho receptor via the portal vein, inhibiting the generation of CYP7A1, thereby reducing BAs synthesis in the liver. CYP7A1 serves as the rate-limiting enzyme for BAs synthesis in classical way; CYP27A1 is the vital enzyme for alternative synthesis of CDCA; Primary BAs are mainly transported by BSEP to the gallbladder for storage.

Modern research has indicated that BAs regulate and activate multiple host metabolic pathways through FXR. The metabolic pathways regulated by FXR mainly include glucose, sterol metabolism, and lipid, and are believed to have the potential to improve conditions such as obesity, liver damage, and chronic inflammatory diseases (Xiang et al., 2021). Several studies have indicated that FXR activation contributes to the improvement of T2DM, and the “BAs − intestinal FXR/FGF15”signaling pathway is an important mechanism through which FXR exerts its effects. An intestinal-restricted FXR agonist (fexaramine) has been found to promote browning of mouse adipose tissue, reducing obesity, inflammation, and insulin resistance (Shapiro et al., 2018). Activation of intestinal FXR can induce the release of FGF15, which reaches liver cells via the portal vein. Then, FGF15 is bound with the liver FGF4/βKlotho receptor, inhibiting the expression of CYP7A1, thereby suppressing the synthesis of BAs in the liver (Liu et al., 2023). The specific mechanism of anti-diabetes is shown in Figure 7. Other studies have found that FGF15/19 released from the small intestine inhibits hepatic gluconeogenesis by suppressing the CREB-PGC-1α pathway (Potthoff et al., 2011). Furthermore, research has suggested that sleeve gastrectomy (SG) serves as a preferred surgical approach by surgeons for treating diabetes mellitus, typically ameliorating hepatic glucose metabolism and improving T2DM via the intestinal-liver crosstalk mediated by FGF15. Following SG, there is an elevation in BAs, which can activate the FXR/FGF15 pathway and then specifically stimulate hepatic FGF4 and its corresponding signaling pathways, promoting hepatic glycogen synthesis, and inhibiting gluconeogenesis (Wei et al., 2023).

This study indicates that JNDX noticeably improves the dysbiosis of gut microbiota and affects BAs (CA and UDCA) metabolism in T2DM rats, both of which have been shown in modern research to activate the “BAs–FXR/FGF15”pathway, thereby improving T2DM. A study has also indicated that increased expression of FXR and FGF15 in the intestine, along with decreased expression of CYP7A1 in the liver, contributed to treating T2DM in rats (Tawulie et al., 2023), which was consistent with the findings of this research. In conclusion, this study reasonably confirms that JNDX treatment for T2DM can exert its effects by regulating the BAs metabolism and FXR/FGF15 signaling.

## 5 Conclusion

Our study firstly explored the potential mechanisms of JNDX in improving T2DM *in vivo*. The results showed that JNDX could effectively improve insulin resistance, hyperglycemia, hyperlipidemia, and inflammation in T2DM rats. Additionally, the protein and mRNA levels of FXR and FGF15 increased after JNDX treatment, while the expression of CYP7A1 decreased. These results suggest that the mechanism by which JNDX ameliorates T2DM may be related to its improvement of BAs metabolic disorders and activation of FXR/FGF15 pathway. Our findings contribute to a better understanding of the therapeutic effect of JNDX on T2DM and provide a scientific basis for its clinical application and drug development.

## Figures and Tables

**Table:** 

**BAs**	bile acids
**BSA**	bovine serum albumin
**BSH**	bile salt hydrolase
**CA**	cholic acid
**CYP7A1**	Cholesterol 7α hydroxylase
**DCA**	deoxycholic acid
**DHCA**	dihydrocaffeic acid
**ELISA**	enzyme-linked immunosorbent assay
**FBG**	fasting blood-glucose
**FGF15**	fibroblast growth factor 15
**FXR**	farnesol X receptor
**GCA**	glycocholic acid
**GLP-1**	glucagon-like peptide 1
**GSP**	glycosylated serum protein
**GUDCA**	glycoursodeoxycholic acid
**HDL-C**	high-density lipoprotein cholesterol
**HFD**	high-fat diet
**HOMA-IR**	ho-meostasis model assessment of insulin resistance
**HPLC-QQQ-MS**	high-performance liquid chromatography-triple quadrupole mass spectrometry
**IL-1β**	interleukin-1β
**IL-6**	interleukin-6
**Ins**	insulin
**ISI**	insulin sensitivity index
**JNDX**	Ji-Ni-De-Xie
**LCA**	lithocholic acid
**LDL-C**	low-density lipoprotein cholesterol
**LPS**	lipopolysaccharide
**mRNA**	messenger RNA
**PVDF**	polyvinylidene difluoride
**RT-qPCR**	real-time quantitative polymerase chain reaction
**SD**	Sprague-Dawley
**SG**	sleeve gastrectomy
**STZ**	streptozotocin
**T2DM**	type 2 diabetes mellitu
**TBST**	Tris Buffered Saline with Tween 20
**TC**	total cholesterol
**TCA**	taurocholic acid
**TCDCA**	taurochenodeoxycholic acid
**TDCA**	taurodeoxycholic acid
**TG**	triglyceride
**TNF-α**	tumor necrosis factor-α
**TUDCA**	tauroursodeoxycholic acid
**T-α-MCA**	tauro-alpha-muricholic acid
**T-β-MCA**	tauro-beta-muricholic acid
**UDCA**	ursodeoxycholic acid
**UPLC-Q-Exactive Orbitrap MS**	ultra high performance liquid chromatography with four stage rod/electrostatic field orbital trap high resolution mass spectrometry

## Data Availability

The data presented in the study are deposited in the NCBI repository (https://www.ncbi.nlm.nih.gov/), accession number PRJNA1110576.
