# Supplementary material for: Ji-Ni-De-Xie ameliorates type 2 diabetes mellitus by modulating the bile acids metabolism and FXR/FGF15 signaling pathway
Source: Front Pharmacol. 2024 May 21;15:1383896. doi: 10.3389/fphar.2024.1383896 (PMC11148236; doi:10.3389/fphar.2024.1383896)
Supplement: Supplementary file 1 [file DataSheet1.docx]

Supplementary Material

# Supplementary Figures and Tables

## Supplementary Figures


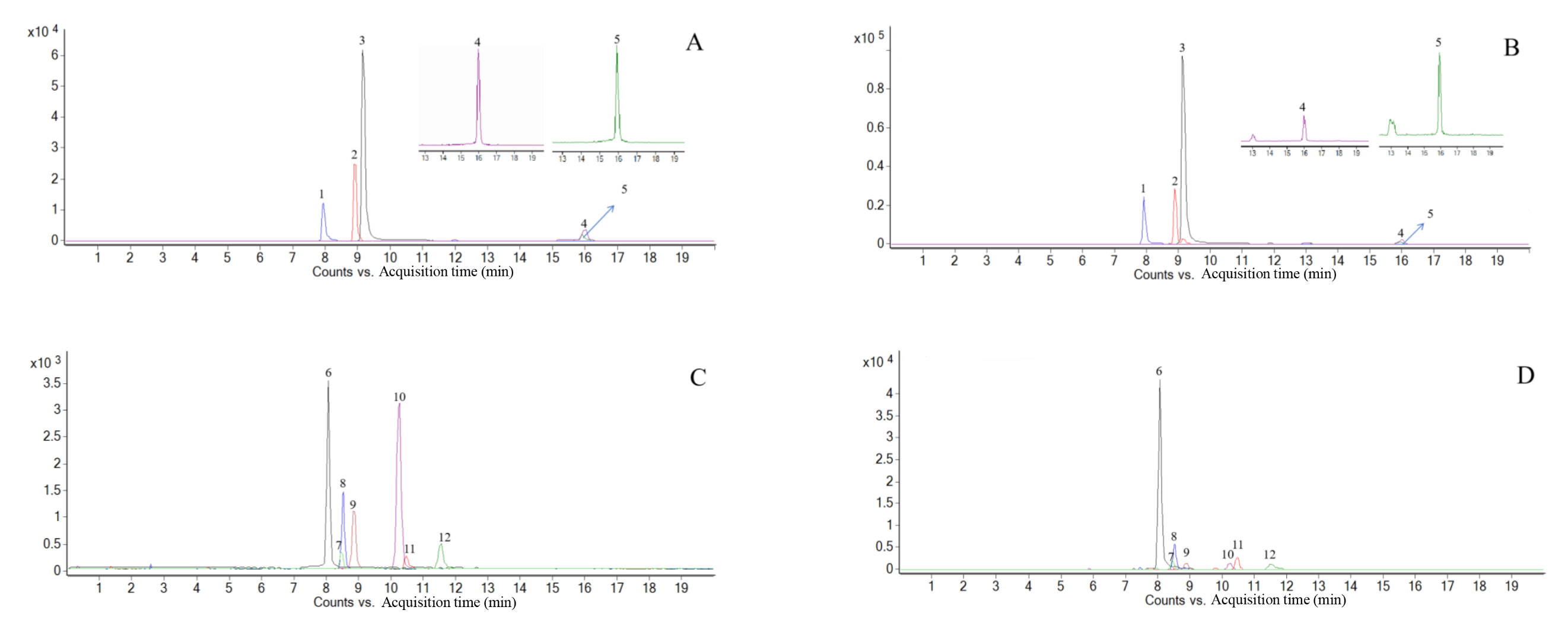


**Figure S1.** Chromatogram of reference (A) and sample (B) in positive ion mode; Chromatogram of reference (C) and sample (D) in negative ion model. (1. Magnoflorine; 2. Jateorrhizine; 3. Berberine; 4. Curcumin; 5. Demethoxycurcumin; 6. Gallic acid; 7. Chebulagic acid; 8. Ferulic acid 4-O-β-D-glucopyranoside; 9. Hydroxysafflor yellow A; 10. Rutin; 11. Ferulic acid; 12. Ellagic acid).


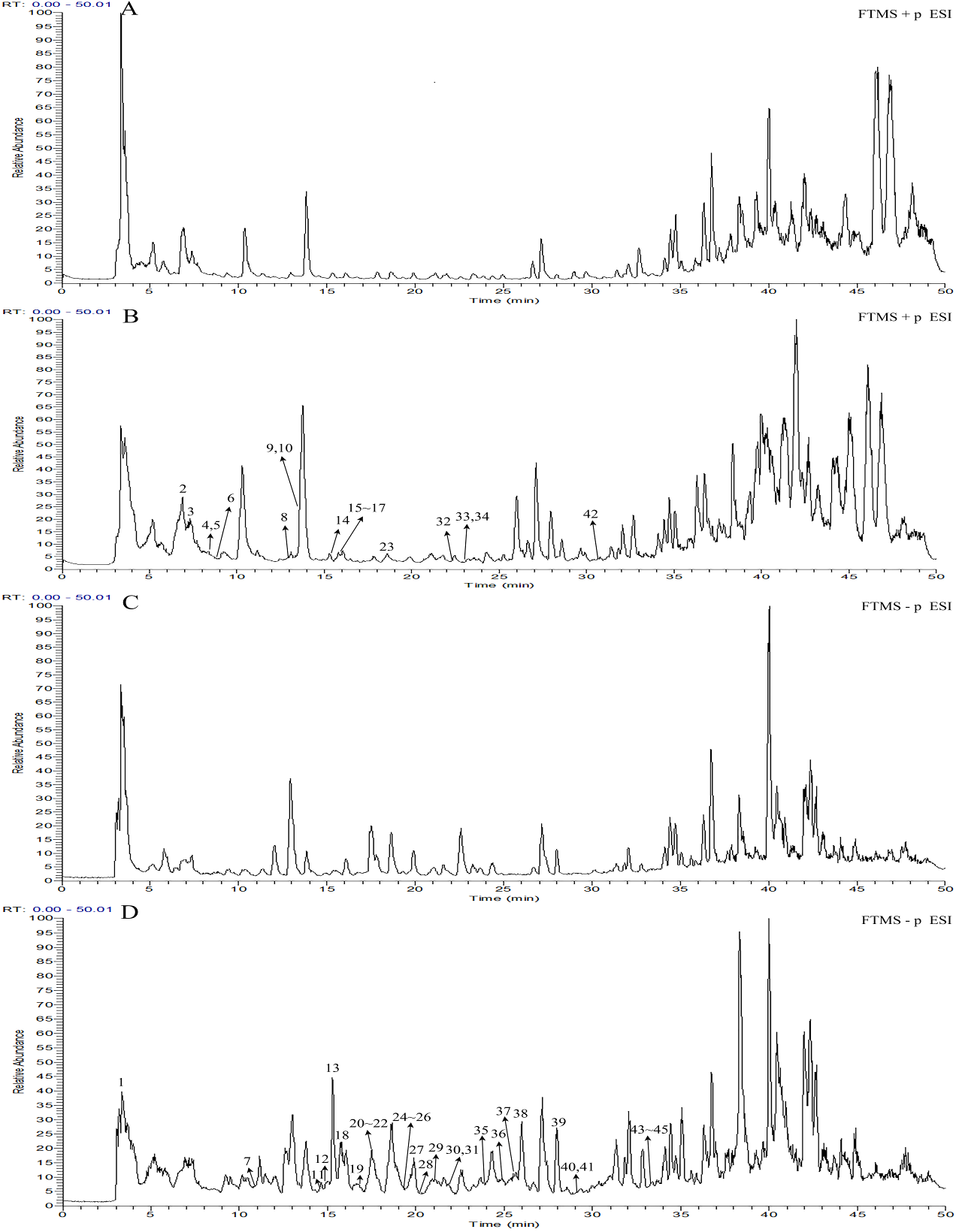
 Figure **Figure S2.** The diagram of positive and negative ion flow (A and C are normal rat plasma; B and D are JNDX rat plasma)

**
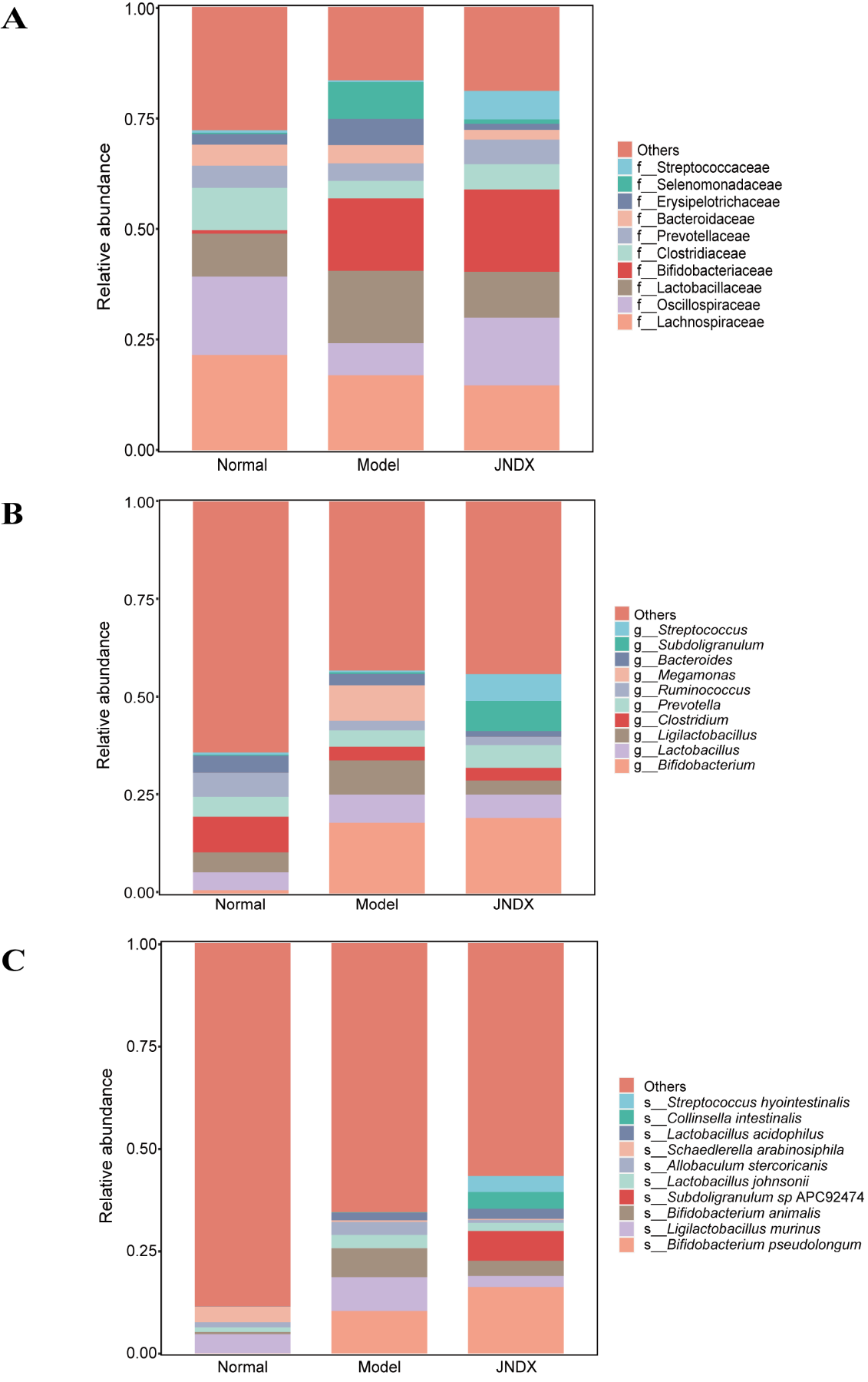
**

**Figure S3** Relative abundance of gut microbiota at the level of (A) family, (B) genus, and (C) species


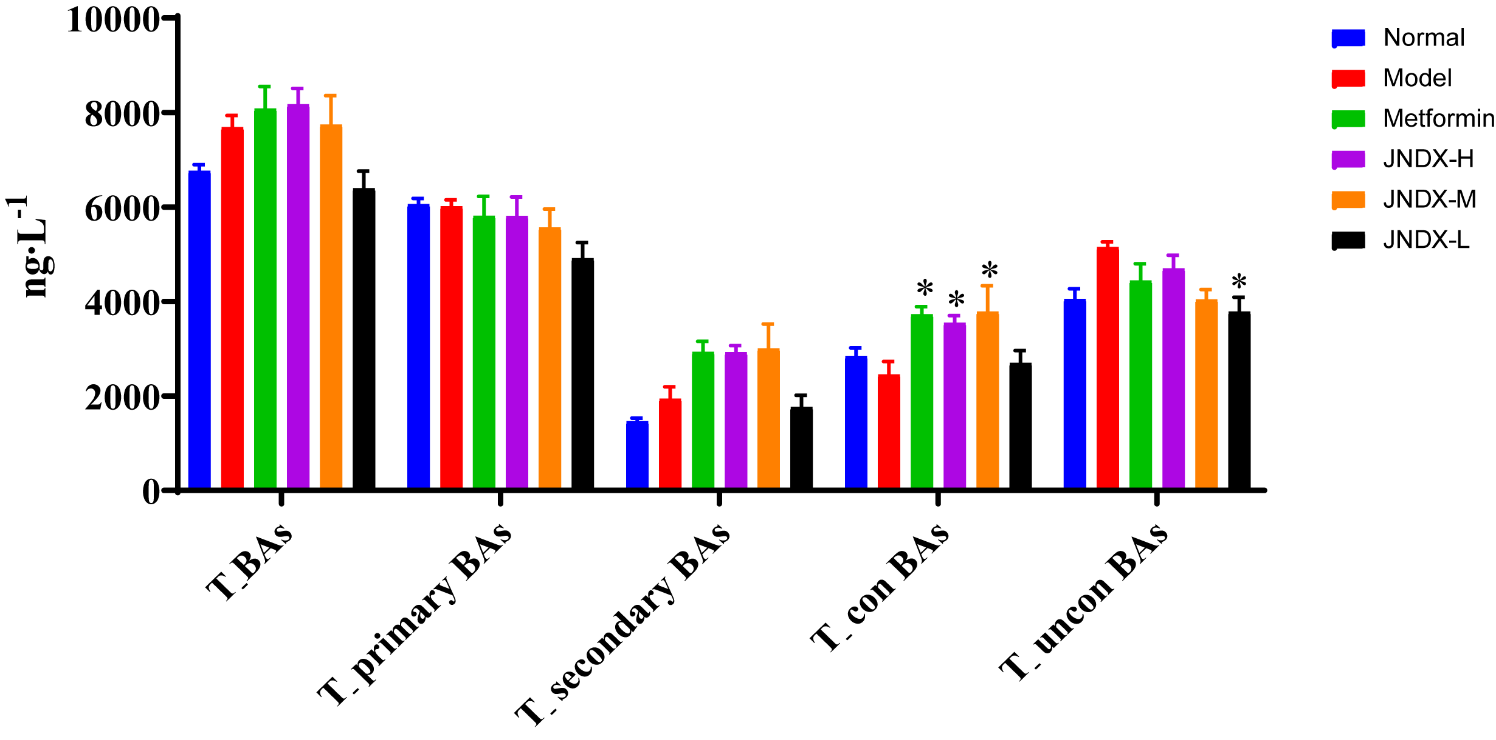
**Figure S4.** Changes in total BAs, total primary BAs, total secondary BAs, total conjugated BAs, and total unconjugated BAs

## Supplementary Tables

**Table S1** Mass spectrum information for 12 chemical components.

| Compound name | Parent ion (m/z) | Daughter ions (m/z) | Rupture voltage  F(V) | Collision energy  CE (eV) | Adducts | t_R_/min |
| --- | --- | --- | --- | --- | --- | --- |
| Magnoflorine | 342.1 | 264.9 | 130 | 24 | [M+H]^+^ | 7.96 |
| Jateorrhizine | 338 | 322 | 140 | 27 | [M+H]^+^ | 8.94 |
| Berberine | 336 | 320.1 | 135 | 27 | [M+H]^+^ | 9.21 |
| Curcumin | 369.4 | 177.1 | 83 | 23 | [M+H]^+^ | 15.98 |
| Demethoxycurcumin | 339.1 | 146.8 | 160 | 19 | [M+H]^+^ | 15.93 |
| Gallic acid | 169 | 124.9 | 120 | -12 | [M-H]^-^ | 8.06 |
| Chebulagic acid | 953.1 | 301 | 270 | -36 | [M-H]^-^ | 8.46 |
| Ferulic acid 4-O-β-D-glucopyranoside | 355.1 | 193.2 | 130 | -6 | [M-H]^-^ | 8.52 |
| Hydroxysafflor yellow A | 611.1 | 490.7 | 160 | -32 | [M-H]^-^ | 8.80 |
| Rutin | 609.1 | 299.9 | 220 | -34 | [M-H]^-^ | 10.19 |
| Ferulic acid | 193 | 134 | 90 | -10 | [M-H]^-^ | 10.42 |
| Ellagic acid | 300.9 | 144.9 | 170 | -40 | [M-H]^-^ | 11.44 |

**Table S2** The content of 12 chemical components in JNDX (mg∙g^-1^).

| Compound name | Content |
| --- | --- |
| Magnoflorine | 3.27 |
| Jateorrhizine | 0.25 |
| Berberine | 1.80 |
| Curcumin | 0.44 |
| Demethoxycurcumin | 0.17 |
| Gallic acid | 18.11 |
| Chebulagic acid | 4.98 |
| Ferulic acid 4-O-β-D-glucopyranoside | 3.07 |
| Hydroxysafflor yellow A | 0.06 |
| Rutin | 0.20 |
| Ferulic acid | 1.83 |
| Ellagic acid | 3.17 |

**Table S3** Identification of constituents derived from JNDX in rat serum

| **No.** | **t_R_/min** | **Formula** | **ion mode** | **Calculated mass (Da)** | **Theoretical (Da)** | **Error (ppm)** | **Secondary fragment ion information** | **Component name** | **Prototype/metabolic components** |
| --- | --- | --- | --- | --- | --- | --- | --- | --- | --- |
| 1 | 3.56 | C_7_H_12_O_6_ | - | 191.0557 | 191.0561 | -2.09 | 191.0557 (100); 173.0452 (2); 127.0395 (4); 93.0337 (6); 87.0079 (4); 85.0284 (20); 59.0127 (3) | Quinic acid | Prototype components |
| 2 | 6.95 | C_19_H_27_N_2_O_7_ | + | 395.1816 | 395.1813 | 0.76 | 395.1816 (28); 336.1073 (16); 219.1494 (12); 160.0759 (100); 132.0810 (2); 115.0545 (1); 60.0814 (7) | Bufotenidine glucuronide | Metabolic components |
| 3 | 7.20 | C_18_H_24_N_2_O_7_ | + | 381.1660 | 381.1656 | 1.05 | 381.1660 (23); 336.1078 (4); 205.1339 (41); 160.0756 (40); 58.0658 (100) | Bufotenine glucuronide | Metabolic components |
| 4 | 8.37 | C_13_H_19_N_2_O | + | 219.1495 | 219.1492 | 1.37 | 219.1495 (11); 160.0759 (100); 159.0679 (1); 132.0810 (4); 115.0547 (2) | Bufotenidine | Prototype components |
| 5 | 8.43 | C_10_H_9_NO | + | 160.0759 | 160.0757 | 1.25 | 160.0759 (100);132.0809 (14); 117.0580 (5); 115.0548 (9) | Bufotenidine N-demethylated metabolite | Metabolic components |
| 6 | 8.90 | C_12_H_16_N_2_O | + | 205.1342 | 205.1355 | -6.34 | 205.1342 (8); 160.0758 (59);132.0810(3); 117.0579(1); 115.0547 (1); 58.0659 (100) | Bufotenine* | Prototype components |
| 7 | 10.98 | C_9_H_10_SO_8_ | - | 277.0023 | 277.0024 | -0.36 | 277.0026 (43); 197.0452 (85);182.0216 (100); 169.0137 (13) | Di-O-methyl ellagic acid sulfate ester | Metabolic components |
| 8 | 13.02 | C_19_H_20_NO_6_ | + | 358.1291 | 358.1285 | 1.68 | 358.1291 (15); 340.1182 (100); 309.0997 (18); 308.0959 (3); 294.0763 (28); 280.0969 (3); 266.0819 (2) | Dihydroxylated demethyleneberberine | Metabolic components |
| 9 | 13.72 | C_9_H_10_O_7_S | + | 261.0079 | 261.0074 | 1.92 | 261.0079 (60); 181.0503 (100); 137.0601 (42); 122.0364 (2); 79.9563 (11) | Ferulic acid demethylation and reduced sulfate conjugate | Metabolic components |
| 10 | 13.77 | C_19_H_22_NO_4_ | + | 328.1546 | 328.1543 | 0.91 | 328.1546 (100); 283.0970 (15); 265.0860 (82); 251.0705 (10); 237.0914 (8); 223.0756 (7); 191.0860 (10); 58.0659 (48) | Demethylated magnoflorine | Metabolic components |
| 11 | 14.55 | C_10_H_12_O_7_S | - | 275.0233 | 275.0231 | 0.73 | 275.0233 (28); 195.0660 (100); 180.0425 (94); 134.0361 (4) | Reduced sulfate conjugate of ferulic acid's metabolite | Metabolic components |
| 12 | 14.82 | C_16_H_18_O_10_ | - | 369.0826 | 369.0827 | -0.27 | 369.0832 (2); 193.0501 (100); 178.0267 (60); 149.0602 (23); 134.0366 (81); 113.0236 (60) | Ferulic acid glucuronide | Metabolic components |
| 13 | 15.31 | C_10_H_10_O_7_S | - | 273.0078 | 273.0074 | 1.47 | 273.0078 (18); 193.0503 (100); 178.0267 (51); 149.0598 (21); 139.0392 (3); 137.0237 (10); 134.0366 (58); 96.9593 (8) | Ferulic acid sulfate conjugate | Metabolic components |
| 14 | 15.32 | C_12_H_15_N_2_O_2_ | + | 219.1133 | 219.1128 | 2.28 | 219.1133 (3); 160.0758 (100); 159.0683 (1); 132.0809 (6); 117.0575 (2); 115.0546 (4) | Bufotenidine demethylenation product | Metabolic components |
| 15 | 15.72 | C_18_H_19_NO_4_ | + | 314.1390 | 314.1387 | 0.95 | 314.1390 (21); 297.1126 (100); 282.0889 (27); 265.0862 (62) | Nordimethyl magnoflorine | Metabolic components |
| 16 | 15.75 | C_20_H_24_NO_4_ | + | 342.1703 | 342.1700 | 0.88 | 342.1703 (100); 297.1125 (39); 282.0887 (12); 265.0862 (29); 237.0913 (10); 191.0859 (6); 58.0658 (71) | Magnoflorine* | Prototype components |
| 17 | 15.80 | C_19_H_21_NO_4_ | + | 328.1544 | 328.1543 | 0.3 | 328.1544 (95); 297.1125 (100); 282.0889 (31); 265.0862 (73); 237.0914 (21); 219.0804 (10); 191.0856 (19) | Demethylated isocorydine | Prototype components |
| 18 | 15.82 | C_10_H_10_O_4_ | - | 193.0504 | 193.0506 | -1.04 | 193.0504 (35); 178.0267 (75); 149.0602 (19); 139.0391 (7); 137.0238 (21); 134.0367 (100); 121.0288 (3) | Ferulic acid* | Prototype components |
| 19 | 16.72 | C_7_H_6_O_4_ | - | 153.0188 | 153.0193 | -3.27 | 153.0188 (91); 109.0289 (100);91.0181 (5) | 2,3-dihydroxybenzoic acid | Prototype components |
| 20 | 17.13 | C_7_H_6_O_4_ | - | 153.0187 | 153.0193 | -3.92 | 153.0187 (87); 109.0286 (100);91.0180 (5) | Protocatechuic acid | Prototype components |
| 21 | 17.25 | C_9_H_10_SO_8_ | - | 277.0023 | 277.0024 | -0.36 | 277.0023 (18);197.0453 (100); 169.0137 (18); 125.0236 (11) | Methyl gallate sulfation | Metabolic components |
| 22 | 17.42 | C_11_H_16_O_6_S | - | 275.0594 | 275.0595 | -0.36 | 275.0594 (71); 195.1024 (46); 180.0786 (100); 79.9563 (55) | 4-(3-hydroxybutyl)-2-methoxyphenyl sulfate ester | Metabolic components |
| 23 | 18.69 | C_26_H_28_NO_10_ | + | 514.1716 | 514.1708 | 1.56 | 514.1716 (44); 338.1390 (100); 323.1155 (28); 322.1076 (28); 308.0921 (14); 294.1126 (21); 280.0975 (5); 279.0899 (5) | Jateorrhizine glucuronide | Metabolic components |
| 24 | 19.43 | C_8_H_8_O_5_ | - | 183.0295 | 183.0288 | 3.82 | 183.0295 (91); 168.0187 (100); 139.0394 (47); 124.0158 (77); | Methyl gallate | Prototype components |
| 25 | 19.46 | C_10_H_12_O_6_S | - | 259.0283 | 259.0282 | 0.39 | 259.0283 (74);179.0708 (100);164.0473 (47); 79.9563 (43) | Curcumin reduced oxidation sulfation | Metabolic components |
| 26 | 19.64 | C_8_H_6_O_4_ | - | 165.0184 | 165.0182 | 1.21 | 165.0184 (7); 121.0286 (100) | m-phthalic acid | Prototype components |
| 27 | 19.96 | C_10_H_12_SO_8_ | - | 291.0183 | 291.0180 | 1.03 | 291.0183 (30); 211.0610 (30); 196.0374 (100) | Ethyl gallate sulfation and methylation | Metabolic components |
| 28 | 20.43 | C_19_H_16_O_10_ | - | 403.0677 | 403.0671 | 1.49 | 403.0677 (4); 227.0349 (100); 175.0243 (7) | Uric acid A glucuronidation product | Metabolic components |
| 29 | 21.19 | C_9_H_8_O_3_ | - | 163.0394 | 163.0390 | 2.45 | 163.0395 (10); 162.8389 (1); 119.0494 (100); 93.0337 (1) | 3-coumaric acid | Prototype components |
| 30 | 22.04 | C_13_H_8_O_7_S | - | 306.9918 | 306.9918 | 0 | 306.9918 (14); 227.0348 (100); 183.0447 (2); 102.9560 (4) | Uric acid A sulfation product | Metabolic components |
| 31 | 22.07 | C_11_H_14_O_6_S | - | 273.0437 | 273.0438 | -0.37 | 273.0437 (100); 193.0865 (69); 79.9565 (28) | 4-(4-hydroxy-3-methoxyphenyl)-butan-2-one sulfate ester | Metabolic components |
| 32 | 22.17 | C_20_H_20_NO_4_ | + | 338.1390 | 338.1387 | 0.89 | 338.1390 (100); 323.1155 (31); 322.1078 (36); 308.0920 (14); 294.1127 (23); 280.0972 (11) | Jateorrhizine* | Prototype components |
| 33 | 23.05 | C_20_H_18_NO_4_^+^ | + | 336.1232 | 336.1230 | 0.6 | 336.1232 (100); 321.1001 (25); 320.0921 (43); 306.0764 (13); 304.0973 (10); 292.0970 (35); 278.0813 (13) | Berberine* | Prototype components |
| 34 | 23.17 | C_21_H_22_NO_4_^+^ | + | 352.1548 | 352.1543 | 1.42 | 352.1548 (100); 337.1313 (22); 322.1077 (14); 308.1285 (22); 294.1129 (15) | Palmatine* | Prototype components |
| 35 | 24.00 | C_10_H_12_O_5_S | - | 243.0333 | 243.0333 | 0 | 243.0333 (49); 163.0760 (46); 148.0524 (100); 146.9610 (57); 118.9655 (16); 79.9564 (42) | 4-(4-hydroxyphenyl)-butan-2-one sulfate ester | Metabolic components |
| 36 | 25.19 | C_14_H_6_O_8_ | - | 300.9992 | 300.9990 | 0.66 | 300.9992 (100); 283.9958 (6); 257.0092 (3); 229.0141 (6); 201.0185 (3); 185.0239 (3) | Ellagic acid* | Prototype components |
| 37 | 25.63 | C_7_H_6_O_3_ | - | 137.0237 | 137.0244 | -5.11 | 137.0237 (35); 93.0337 (100) | 4-Hydroxybenzoic acid | Metabolic components |
| 38 | 26.31 | C_9_H_16_O_4_ | - | 187.0972 | 187.0976 | -2.14 | 187.0972 (65); 169.0860 (3); 125.0964 (100) | Azelaic acid | Prototype components |
| 39 | 28.21 | C_15_H_10_O_7_ | - | 301.0353 | 301.0343 | 3.32 | 301.0353 (100); 178.9975 (25); 151.0029 (74); 121.02878 (32); 107.0129 (22) | Quercetin* | Prototype components |
| 40 | 29.19 | C_16_H_10_O_8_ | - | 329.0305 | 329.0303 | 0.61 | 329.0305 (90); 314.0071 (84); 312.9990 (42); 298.9836 (100) | Dimethyl ellagic acid | Metabolic components |
| 41 | 29.22 | C_8_H_8_O_3_ | - | 151.0395 | 151.0390 | 3.31 | 151.0395 (34); 136.0158 (11); 123.0443 (5); 107.0493 (100) | Vanillin | Prototype components |
| 42 | 30.64 | C_15_H_20_O | + | 217.1592 | 217.1587 | 2.3 | 217.1592 (43); 119.0860 (100); 91.0549 (7) | Ar-turmerone | Prototype components |
| 43 | 33.28 | C_19_H_16_O_4_ | - | 307.0970 | 307.0976 | -1.95 | 307.0970 (2); 187.0399 (17); 143.0495 (33); 119.0494 (100) | Bis-demethoxycurcumin* | Prototype components |
| 44 | 33.36 | C_20_H_18_O_5_ | - | 337.1075 | 337.1081 | -1.78 | 337.1075 (2); 217.0506 (15); 202.0270 (5); 173.0603 (12); 149.0601 (10); 119.0494 (100) | Demethoxycurcumin* | Prototype components |
| 45 | 33.46 | C_21_H_20_O_6_ | - | 367.1196 | 367.1187 | 2.45 | 367.1196 (2); 217.0505 (24); 202.0267 (5); 173.0603 (23); 149.0602 (57) | Curcumin* | Prototype components |

**Table S4** Relative abundance composition of gut microbiota at the 3 levels

| Taxonomic hierarchy | Bacteria | Normal | Model | JNDX |
| --- | --- | --- | --- | --- |
| Family | Lachnospiraceae | 21.503% | 16.900% | 14.600% |
|  | Oscillospiraceae | 17.657% | 7.217% | 15.335% |
|  | Lactobacillaceae | 9.695% | 16.401% | 10.324% |
|  | Bifidobacteriaceae | 0.778% | 16.313% | 18.581% |
|  | Clostridiaceae | 9.603% | 3.949% | 5.681% |
|  | Prevotellaceae | 4.971% | 3.966% | 5.590% |
|  | Bacteroidaceae | 4.738% | 4.149% | 2.203% |
|  | Erysipelotrichaceae | 2.426% | 5.899% | 1.322% |
|  | Selenomonadaceae | 0.280% | 8.251% | 0.998% |
|  | Streptococcaceae | 0.535% | 0.411% | 6.472% |
|  | Others | 27.813% | 16.544% | 18.893% |
| Genus | *Bifidobacterium* | 0.838% | 17.998% | 19.253% |
|  | *Lactobacillus* | 4.552% | 7.201% | 5.967% |
|  | *Ligilactobacillus* | 5.074% | 8.732% | 3.617% |
|  | *Clostridium* | 9.133% | 3.524% | 3.208% |
|  | *Prevotella* | 5.060% | 4.149% | 5.778% |
|  | *Ruminococcus* | 6.073% | 2.446% | 2.099% |
|  | *Megamonas* | 0.017% | 9.090% | 0.033% |
|  | *Bacteroides* | 4.426% | 2.910% | 1.429% |
|  | *Subdoligranulum* | 0.226% | 0.390% | 7.689% |
|  | *Streptococcus* | 0.553% | 0.415% | 6.889% |
|  | Others | 64.049% | 43.145% | 44.038% |
| Species | *Bifidobacterium pseudolongum* | 0.062% | 10.357% | 16.185% |
|  | *Ligilactobacillus murinus* | 4.612% | 8.214% | 2.721% |
|  | *Bifidobacterium animalis* | 0.585% | 7.027% | 3.736% |
|  | *Subdoligranulum sp* APC92474 | 0.039% | 0.037% | 7.199% |
|  | *Lactobacillus johnsonii* | 1.094% | 3.234% | 1.981% |
|  | *Allobaculum stercoricanis* | 1.213% | 3.148% | 0.729% |
|  | *Schaedlerella arabinosiphila* | 3.760% | 0.431% | 0.274% |
|  | *Lactobacillus acidophilus* | 0.047% | 1.871% | 2.404% |
|  | *Collinsella intestinalis* | 0.020% | 0.064% | 4.099% |
|  | *Streptococcus hyointestinalis* | 0.001% | 0.026% | 3.931% |
|  | Others | 88.567% | 65.591% | 56.741% |
